# Ezh2-dCas9 and KRAB-dCas9 enable engineering of epigenetic memory in a context-dependent manner

**DOI:** 10.1186/s13072-019-0275-8

**Published:** 2019-05-03

**Authors:** Henriette O’Geen, Sofie L. Bates, Sakereh S. Carter, Karly A. Nisson, Julian Halmai, Kyle D. Fink, Suhn K. Rhie, Peggy J. Farnham, David J. Segal

**Affiliations:** 1 Genome Center and Department of Biochemistry and Molecular Medicine, University of California, Davis, CA 95616 USA; 2 Department of Biochemistry and Molecular Medicine, Norris Comprehensive Cancer Center, Keck School of Medicine, University of Southern California, Los Angeles, CA 90089 USA; 3 Department of Neurology and Stem Cell Program, University of California, Sacramento, CA 95817 USA

**Keywords:** Epigenetic memory, Epigenome editing, CRISPR–dCas9, Ezh2, Epigenetics, Histone methylation, DNA methylation, Chromatin, Gene expression, Off-target effects

## Abstract

**Background:**

Rewriting of the epigenome has risen as a promising alternative to gene editing for precision medicine. In nature, epigenetic silencing can result in complete attenuation of target gene expression over multiple mitotic divisions. However, persistent repression has been difficult to achieve in a predictable manner using targeted systems.

**Results:**

Here, we report that persistent epigenetic memory required both a DNA methyltransferase (DNMT3A-dCas9) and a histone methyltransferase (Ezh2-dCas9 or KRAB-dCas9). We demonstrate that the histone methyltransferase requirement can be locus specific. Co-targeting Ezh2-dCas9, but not KRAB-dCas9, with DNMT3A-dCas9 and DNMT3L induced long-term *HER2* repression over at least 50 days (approximately 57 cell divisions) and triggered an epigenetic switch to a heterochromatic environment. An increase in H3K27 trimethylation and DNA methylation was stably maintained and accompanied by a sustained loss of H3K27 acetylation. Interestingly, substitution of Ezh2-dCas9 with KRAB-dCas9 enabled long-term repression at some target genes (e.g., *SNURF*) but not at *HER2*, at which H3K9me3 and DNA methylation were transiently acquired and subsequently lost. Off-target DNA hypermethylation occurred at many individual CpG sites but rarely at multiple CpGs in a single promoter, consistent with no detectable effect on transcription at the off-target loci tested. Conversely, robust hypermethylation was observed at *HER2*. We further demonstrated that Ezh2-dCas9 required full-length DNMT3L for maximal activity and that co-targeting DNMT3L was sufficient for persistent repression by Ezh2-dCas9 or KRAB-dCas9.

**Conclusions:**

These data demonstrate that targeting different combinations of histone and DNA methyltransferases is required to achieve maximal repression at different loci. Fine-tuning of targeting tools is a necessity to engineer epigenetic memory at any given locus in any given cell type.

**Electronic supplementary material:**

The online version of this article (10.1186/s13072-019-0275-8) contains supplementary material, which is available to authorized users.

## Introduction

Epigenome engineering, the targeted rewriting of epigenetic information, has risen as a promising alternative to cleavage-dependent gene editing, but our knowledge related to engineering long-term epigenetic memory is limited (reviewed in [1, 2]). Distinct epigenomic profiles define cellular identity among cells carrying the same genetic information. Chromatin organization is tightly regulated during development and is critical for establishing and maintaining cell-type-specific transcriptional programs forming the foundation of cellular memory. Acquired epigenetic alterations can lead to aberrant epi-alleles implicated in diseases, such as cancers, cardiovascular disease and mental disorders [3, 4].

Epigenetic marks, including posttranslational histone modifications and DNA methylation, have been profiled across hundreds of human tissues, and cells have provided maps of unique cellular identity [5, 6]. Transcriptional activity has been associated with certain epigenetic marks. For example, repressed genes have been associated with posttranslational modifications (PTM) on histone tails including trimethylation of lysines 9 and 27 on histone H3 (H3K9me3 and H3K27me3, respectively) and ubiquitination of histone H2A on lysine 119 (H2AK119u1) [7–9]. Although these are all hallmarks of a repressive chromatin state, they mark distinct regions of the genome. H3K9me3 is typically present at constitutive heterochromatin associated with repeat regions of the genome, while H3K27me3 and H2AK119u1 mark facultative heterochromatin and the inactive X chromosome and play a critical role at developmentally regulated genes [8, 10–13]. The patterns of histone marks and chromatin-associated proteins define distinct transcriptional states that are established and maintained through a dynamic interplay of histone readers, writers and erasers creating a positive feedback loop. For example, trimethylation of H3K27 is mainly maintained by one enzyme, namely Enhancer of Zeste Homolog 2 (EZH2). EZH2 is the catalytic subunit of the Polycomb Repressive Complex 2 (PRC2), which also contains SUZ12 and EED [14, 15]. EED mediates PRC2 binding to H3K27me3 and is required for the propagation of H3K27me3 domains, suggesting a mechanism for the inheritance of the H3K27me3 mark through cell division [8]. DNA methylation on carbon 5 of cytosine (5mC) in promoter regions has also been associated with gene repression [16, 17]. DNMT3A and DNMT3B provide de novo DNA methylation in conjunction with the non-catalytic DNMT3L binding partner, while maintenance of DNA methylation is provided by DNMT1. Heterochromatin marked by both H3K9me3 and 5mC is bound by the KAP1/SETDB1(ESET) co-repressor complex, which is recruited by the Krueppel-associated box (KRAB) domain [18, 19]. The KAP1/SETDB1 complex also recruits HP1 and DNMT3A.

Several tools have been developed to repress or activate gene expression or modulate epigenetic marks at a defined locus. These epigenetic editing tools consist of a DNA binding module and an epigenetic effector domain. The easily programmable CRISPR/Cas9 system is the method of choice for precise genome targeting. Two single amino acid changes converts the Cas9 nuclease into a catalytically inactive or “dead” Cas9 (dCas9), which associates with its DNA target sequence through simple base pairing of a short RNA guide to which it is bound [20]. Epigenetic effector domains have been fused to dCas9 (epi-dCas9), which allow precise targeting of promoter and/or enhancer regions to alter associated epigenetic marks, typically with the readout of gene activation or repression [2]. The KRAB domain is the most commonly used repression domain [20–22]. Because of its potent repressive capacity, dCas9-KRAB fusions are now used for high-throughput gene discovery screens [23, 24]. However, the repressive activity of KRAB-dCas9 fusions at the target locus is only of transient nature and transcription is re-established once the transcriptional modifier protein is depleted [25, 26]. For example, we have previously reported that targeting the *HER2* (*ERBB2*) gene with epi-dCas9 fusions to KRAB, FOG1, Ezh2 and DNMT3A can induce transient repression of *HER2* gene expression in HCT116 cells [26]. As expression of epi-dCas9 subsided, *HER2* expression was re-established to original levels.

Unlike the forced epigenetic changes described above, natural epigenetic changes often lead to robust and persistent changes in gene expression, sometimes lasting over the lifetime of an individual. Here, we have investigated the parameters required to achieve persistent epigenetic silencing of gene expression. Tools to engineer epigenetic memory are starting to emerge, but our understanding of the requirements for a persistent epigenetic switch is in its infancy. Others have reported [25, 27] that persistent gene repression requires the combination of KRAB (recruiting a complex containing the histone methylase SETDB1) and a DNA methyltransferase. However, we observed that this combination is not effective in inducing long-term epigenetic silencing at any given locus. In this study, we demonstrate that the combination of DNA methylation with a different histone methyltransferase, namely Ezh2, is necessary to induce a persistent epigenetic switch and long-term repression of the *HER2* oncogene in HCT116 cells. Global methylation analysis in cells in which Ezh2-dCas9 and KRAB-dCas9 was transiently targeted to the *HER2* locus revealed hypermethylation of many individual CpG probes throughout the genome even 3 weeks after exposure, but rarely resulted in differentially hypermethylated regions (DMRs) of > 3 CpGs within gene promoters. Notably, hypermethylation of > 3 promoter CpGs did not result in a change of transcription at the examined off-target loci. However, close investigation of the chromatin state at the *HER2* locus revealed that long-term repression facilitated by Ezh2 and DNA methylases corresponds with an engineered and stably maintained heterochromatic environment of H3K27 trimethylation and DNA methylation. In fact, DNA methylation expanded beyond the genomic target sites, leading to a 1.25-kb hypermethylated region at the *HER2* promoter. We extended our evaluation of inducing long-term repression to two loci in different mouse and human cell lines. We demonstrated that DNA methylation improved long-term silencing by KRAB-dCas9, but was absolutely required for robust long-term repression by Ezh2-dCas9. In summary, our data demonstrate that we can induce a persistent locus-specific epigenetic switch, but different histone and DNA methyltransferases are required to achieve long-term repression at different loci and/or in different cell types.

## Materials and methods

### Plasmids

Plasmids expressing dCas9 fusions with KRAB, Ezh2, DNMT3A effector domains, as well as the dCas9 cloning vector without any effector domain, have previously been described [26] and are available through Addgene (KRAB-dCas9 #112195 Ezh2-dCas9 #100086, dCas9 #100091). The DNMT3L expression plasmid pCDNA-DNMT3L was a kind gift from Dr. Fred Chedin [28]. DNMT3A-dCas9 (Addgene #100090) and pCDNA-DNMT3L are abbreviated in this study to D3A-dCas9 and D3L, respectively. DNMT3L was amplified from pCDNA-DNMT3L with overhangs for Gibson cloning into a KpnI- or NheI-digested dCas9 cloning vector, resulting in D3L-dCas9 (Addgene) and dCas9-D3L (Addgene), respectively. Protein sequences are shown in Additional file 1: Figure S1. A plasmid expressing a hybrid of mouse Dnmt3a and human DNMT3L fused to dCas9 (dCas9-D3a3L) was a kind gift from Dr. Albert Jeltsch [29]. The gRNA cloning vector, plasmid # 41824, was obtained from Addgene [30], and the 19-bp gRNA target sequences were selected within 500 bp of each relevant gene promoter using the online tool CHOPCHOP v2 [31]. Each gRNA sequence was cloned as G-N19 into the AflII-linearized plasmid, as previously described [26]. The gRNA sequences used to create target-specific vectors are listed in Additional file 2: Table S1. Plasmids expressing 1xMCP-effector fusions were generated by cloning effector domains into NcoI- and ClaI-digested UBC-NLS-HA-2XMCP-tagRFPt [32] Addgene plasmid #64541). 2xMCP-effector fusion plasmids were created using XbaI- and ClaI-digested UBC-NLS-HA-2XMCP-tagRFPt. KRAB and Ezh2 effector domains were amplified with overhangs from KRAB-dCas9 and Ezh2-dCas9 and cloned using the Gibson method into the appropriately cut vector. Protein sequences for 2xMCP-KRAB (Addgene) and 2xMCP-Ezh2 (Addgene) are shown in Additional file 1: Figure S1. MS2-gRNA expressing plasmids were created using the sgRNA(MS2) backbone [33], Addgene plasmid #61427) following the authors’ instructions (http://sam.genome-engineering.org/protocols/). MS2-gRNA sequences are listed in Additional file 2: Table S1.

### Cell culture and transfections

HCT116 cells (ATCC #CCL-247) were maintained in McCoy’s 5A medium supplemented with 10% fetal bovine serum (FBS) and 1% penicillin/streptomycin. LNCaP cells (ATCC #CRL-1740) were grown in RPMI-1640 medium supplemented with 10% FBS and 1% penicillin/streptomycin. 3T3 cells were grown in Dulbecco’s modified Eagle’s medium supplemented with 10% FBS and 1% penicillin/streptomycin. Neuro2A (ATCC #CCL-131) cells were maintained in Dulbecco’s modified Eagle’s medium (DMEM) supplemented with 10% fetal calf serum (FCS). All cells were grown at 37 °C under 5% CO_2_. Cells of 60–70% confluency were transfected using Lipofectamine 3000 (Life Technologies) following the manufacturer’s instructions. Transfections for RNA extraction and FACS sorting were performed in 12-well plates using 625 ng individual or combinations of dCas9 expression vector, 500 ng of equimolar pooled gRNA expression vectors. Transfections for MS2-based recruitment were carried out using the same protocol with the following plasmid amounts: 337 ng MS2-gRNA pool, 450 ng dCas9 expression vector and 338 ng MCP-effector plasmid. All experiments were co-transfected with 125 ng of puromycin-resistant plasmid pBABE-puro to select for transfected cells. Transfection medium was replaced 24 h post-transfection with growth medium containing 3-µg/ml puromycin to enrich for transfected cells. After 72 h of puromycin selection, cells for RNA isolation were stored at 4 °C in RNAlater (Ambion) or collected for sorting by flow cytometry. To assay for persistent repression, media was switched to standard growth media 4 days after transfection. For ChIP assays, cells were plated in 10-cm culture dishes and transfection followed the same protocol except that reagents were scaled up accordingly.

### Flow cytometry

Transfected HCT116 cells were assayed by flow cytometry after 72-h puromycin selection (96 h after transfection). For flow cytometry collection of the HER2-negative cell population, cells were harvested from two independent biological replicates, washed once in PBS, and resuspended in 1% bovine serum albumin (BSA) in PBS. After adding 3 µl APC-conjugated antihuman CD340 (erbB2/HER-2) antibody (Biolegend #324408), cells were incubated for 30 min at 4 °C. Labeled cells were washed once and resuspended in 1% BSA in PBS. Cell sorting was performed using an Astrios EQ cell sorter (Beckman Coulter) at the UC Davis Flow Cytometry Shared Resource Core. Untreated HCT116 control cells were used to determine the APC signal for HER2-expressing cells, while unlabeled cells were used to determine the sorting gate for HER2-negative cells. Four days after combinatorial epi-dCas9 transfections, HER2-negative cells were collected and replated using standard media. Cells were harvested at indicated time points, dependent on cells reaching ~ 80% confluency. Cells were expanded in 24-well dishes for RNA isolation (5 days, 14 days, 23 days, 39 days, 44 days and 50 days) and in 6-well dishes for DNA methylation analysis (10 days, 17 days and 24 days) and Western blot analysis (40 days). For ChIP assays (24 days), cells were plated in 10-cm dishes.

### RNA extraction and reverse-transcription quantitative PCR (RT-qPCR)

Cells for RNA isolation were stored in RNAlater (Ambion) at 4 °C for up to 1 week. RNAlater was removed, and total RNA was isolated using the RNeasy Plus RNA isolation kit (Qiagen). RNA was reverse-transcribed using the SuperScript VILO MasterMix (Invitrogen). RT-qPCR was performed in triplicate using 2 × iQ SYBR mix (Bio-Rad) with the CFX384 Real-Time System C1000 Touch system (Bio-Rad). Gene expression analysis was performed with GAPDH as a reference gene using at least three biological replicates and the following primer sequences: human HER2 primers (HER2-F 5′-GGGAAACCTGGAACTCACCT-3′; HER2-R 5′-GACCTGCCTCACTTGGTTGT-3′), SNURF (human) primers (SNURF-F 5′-CTGTCTGAGGAGCGGTCAGT-3′; SNURF-R 5′-CAGGTACTTGCTGCTGCTGA-3′), human GAPDH primers (GAPDH-F 5′-AATCCCATCACCATCTTCCA-3′; GAPDH-R 5′-CTCCATGGTGGTGAAGACG-3′), mouse Snurf primers (mSnurf-F 5′-TTGGTTCTGAGGAGTGATTTGC-3′; mSnurf-R 5′-CCTTGAATTCCACCACCTTG-3′), and mouse Gapdh primers (mGapdh-F 5′-TGACCACAGTCCATGCCATC-3′; mGapdh-R 5′-GACGGACACATTGGGGGTAG-3′). Primer sequences used for RT-qPCR of potential off-targets are listed in Additional file 2: Table S1. Relative target gene expression was calculated as the difference between the target gene and the GAPDH reference gene (dCq = Cq[target] − Cq[GAPDH]). Gene expression results are indicated as fold change to a reference sample (usually dCas9 without any effector domain), using the ddCq method. A one-way ANOVA (ANalysis Of VAriance) with post hoc Tukey HSD (honestly significant difference) or Dunnett’s test was used to determine statistical significance for different epi-dCas9 treatments.

### Western blot analysis

Transfected HER2-negative cells were collected by flow cytometry 4 days after transfection and harvested for protein extraction 40 days after transfection. Cells were lysed in 1 × RIPA buffer (Millipore) supplemented with protease inhibitor cocktail (Roche), and protein concentrations were determined by Bradford assay (Bio-Rad). For Western blots, 30 μg of protein was separated on a 4–15% TGX gel (Bio-Rad) with Tris/glycine/sodium dodecyl sulfate buffer. Protein was subsequently transferred onto a nitrocellulose membrane, and protein loading was evaluated by Ponceau S stain. The membrane was rinsed with deionized water and incubated in blocking solution (5% nonfat dry milk in TBST; 50 mM Tris, 150 mM NaCl, 0.1% Tween-20) for 30 min at room temperature. Membranes were incubated with primary antibody in blocking solution at 4 °C overnight. We used Anti-c-ErbB2/c-Neu (Ab-3) mouse mAb (3B5) antibody at 1:1000 dilution (SIGMA #OP15). After three 10-min washes with TBST, the membrane was incubated with horseradish peroxidase-conjugated antimouse secondary antibody (1:2000 dilution in TBST) for 45 min at room temperature. After three more washes in TBST, proteins were visualized with Amersham ECL Prime Western Blotting Detection Reagent (GE Healthcare) using the ChemiDoc XRS Imaging System (Bio-Rad).

### Chromatin immunoprecipitation (ChIP) and ChIP-qPCR

HCT116 cells were transfected with D3A-dCas9/D3L and either KRAB-dCas9 or Ezh2-dCas9 and three gRNAs targeting the *HER2* promoter. Control cells were transfected with dCas9 and the same three gRNAs. For transient repression timepoints, cells were cross-linked 4 days after transfection and growth in puromycin-containing media. For persistent repression timepoints, cells were sorted as described above and dCas9 control cells were cross-linked 24 days after transfection. Cross-linking was carried out in 1% formaldehyde for 10 min at room temperature and was stopped with 0.125 M glycine. Cross-linked cells were lysed with ChIP lysis buffer (5 mM PIPES pH8, 85 mM KCl, 1% Igepal) with a protease inhibitor (PI) cocktail (Roche). Nuclei were collected by centrifugation at 2000 rpm. for 5 min at 4 °C and lysed in nuclei lysis buffer (50 mM Tris pH8, 10 mM EDTA, 1% SDS) supplemented with PI cocktail. Chromatin was fragmented using the Bioruptor 2000 (Diagenode) and diluted with 5 vol RIPA buffer (50 mM Tris pH 7.6, 150 mM NaCl, 1 mM EDTA pH8, 1% Igepal, 0.25% deoxycholic acid). ChIP enrichment was performed by incubation with 3 µg H3K9me3 antibody (Diagenode C15410056), 2 µg H3K27me3 antibody (MP07–449), 2 µg H3K27ac antibody (Active Motif #39133) or 2 µg normal rabbit IgG (Abcam ab46540) for 16 h at 4 °C. Immune complexes were bound to 20 µl magnetic protein A/G beads (ThermoFisher) for 2 h at 4 °C. Beads were washed 2 × with RIPA and 3 × with ChIP wash buffer (100 mM Tris pH8, 500 mM LiCl, 1% deoxycholic acid). The final wash was performed in ChIP wash buffer with 150 mM NaCl. Cross-links were then reversed by heating beads in 100 µl ChIP elution buffer (50 mM NaHCO_3_, 1% SDS) overnight at 65 °C, and DNA was purified using the QIAquick PCR Purification Kit (Qiagen). qPCR was performed with 2 × SYBR FAST mastermix (KAPA Biosystems) using the CFX384 Real-Time System C1000 Touch Thermo Cycler (Bio-Rad). HER2 ChIP amplification primers are as follows: HER2-ChIP-F (5′-TTGGAATGCAGTTGGAGGGG-3′) and HER2-ChIP-R (5′-GGTTTCTCCGGTCCCAATGG-3′). ChIP enrichment was calculated relative to input samples using the dCq method (dCq = Cq[HER2-ChIP] − Cq[input]). Statistical significance was determined by Student’s *t* test.

### ChIP-sequencing and data analysis

Each entire ChIP sample was used to prepare Illumina sequencing libraries using the KAPA Hyper Prep Kit (Roche) and NEXTflex DNA barcodes (BIOO Scientific). Illumina sequencing libraries were pooled and sequenced using the HiSeq 4000 platform (Illumina) at the UC Davis DNA Technologies Sequencing Core. Short sequence reads (SR50) were aligned to the hg19 genome assembly using using BWA [34], and data were preprocessed using the ENCODE3 ChIP-seq pipeline (https://www.encodeproject.org/chip-seq/). ChIP-seq peaks were called using MACS2 [35] from each replicate first, and then, reproducible peaks were selected using the naïve overlap tool, as suggested in the ENCODE3 ChIP-seq standards document (https://www.encodeproject.org/pages/pipelines/) and previously described [36]. To find reduced H3K27ac ChIP-seq promoter peaks in cells treated with Ezh2 plus D3A and D3L compared to dCas9 control, we selected top 15 k robust reproducible peaks from each dataset. H3K27ac peaks only found in the dCas9 control were identified by overlap analysis with treated cells using bedtools (https://github.com/arq5x/bedtools2). Lastly, peaks located in promoter regions (1-kb windows of transcription start sites) of protein coding genes (Gencode version 28, https://www.gencodegenes.org) were identified (*n* = 469 peaks). Using the DiffBind R package [37], ChIP-seq signals were normalized across samples and fold change of reduced peaks was calculated using average signal values (Additional file 6: Table S2). ChIP-seq data have been submitted to the Gene Expression Omnibus (GEO) and are available under accession number GSE123882.

### Analysis of methylation by bisulfite conversion

Genomic DNA from biological replicates was isolated using the Quick-DNA Miniprep Kit (ZYMO), and bisulfite conversion was performed starting with 500 ng genomic DNA with the EZ DNA Methylation-Gold Kit (ZYMO) following the manufacturer’s instructions. Bisulfite-Sequencing PCR primers were designed using MethPrimer [38] (BSS-HER2-F 5′-GGAGGGGGTAGAGTTATTAGTTTTT-3′ and BSS-HER2-R 5′- CACCTCCTCCTTCTCCTATAATTAAA-3′); 100 ng bisulfite converted DNA was used for PCR amplification with ZymoTaq polymerase (ZYMO), and the 229-bp PCR product was purified with the QIAquick PCR Purification Kit (Qiagen). Amplicons were subcloned into the pCR2.1-TOPO TA vector using the TOPO TA cloning kit (ThermoFisher) and transformed into NEB5α competent cells (NEB). Individual clones were subjected to Sanger sequencing (Genewiz), and methylation status of 11 CpGs immediately upstream of the *HER2* TSS (hg19, chr17:37,856,035–37,856,263) was determined. The same amplicons from bisulfite converted DNA were sent for high-throughput sequencing at the CCIB DNA Core Facility at Massachusetts General Hospital (Cambridge, MA). Sequencing data were processed using FLASH2 to merge overlapping paired-end reads and single long reads were then demultiplexed using FASTX barcode splitter by matching barcodes at the beginning or end of the sequences, with one mismatch allowed. Methylation levels were of bisulfite-treated sequencing reads and were analyzed with Bismark [39] using a bisulfite reference and default settings.

### Global DNA methylation analysis

HCT116 cells were transfected with D3A-dCas9/D3L and either KRAB-dCas9 or Ezh2-dCas9 and three gRNAs targeting the *HER2* promoter. Each replicate (*n* = 2) was performed as an entirely separate transfection experiment. Transfected cells were sorted by flow cytometry as described above and genomic DNA was extracted. Control cells were transfected with three HER2 gRNAs and dCas9 (no effector domain). Genomic DNA from sorted and control cells was isolated 17 and 24 days after transfection using the Quick-gDNA MiniPrep kit (ZYMO). The Infinium Human MethylationEPIC BeadChip (Illumina) was used to analyze global DNA methylation. Genomic DNA was bisulfite converted and EPIC DNA methylation array data were processed in the USC Molecular Genomics Core; the methylation status of over 850,000 CpG probes was reported. For each probe, methylated (*M*) and unmethylated (*U*) signal intensities were recorded and the beta value (*M*/(*M* + *U*)) was determined for each probe. Samples were normalized (background corrected) using the ‘noob’ function in the minfi software program in R computing language. All downstream analysis was conducted using the hg19/GRCh37 human genome assembly. To identify hypermethylated probes located in promoter regions, we first selected probes located in promoter regions (Illumina MethylationEPIC Manifest RefGene annotation 5′UTR, TSS200 and TSS1500) (511,700 CpG probes), and then, probes showing increased DNA methylation levels in treated cells (KRAB + D3A + D3L 17 days, Ezh2 + D3A + D3L 17 days, and Ezh2 + D3A + D3L 24 days) compared to dCas9 control samples (mean beta value difference cut off 0.2) were selected (223, 2966, 2018 probes for KRAB + D3A + D3L 17 days, Ezh2 + D3A + D3L 17 days, and Ezh2 + D3A + D3L 24 days, respectively; Additional file 9: Table S3). On the other hand, hypomethylated probes located in promoter regions were identified by selecting probes showing decreased DNA methylation levels in treated samples compared to control samples (mean beta value difference cut off 0.2). Genes that include more than 3 hypermethylated promoter probes were selected to generate Venn diagrams. EPIC array data have been submitted to the Gene Expression Omnibus (GEO) and are available under accession number GSE123830.

## Results

### Ezh2-dCas9, but not KRAB-dCas9, enables long-term silencing by creating a persistent heterochromatin environment at the *HER2* locus

**Fig. 1:**
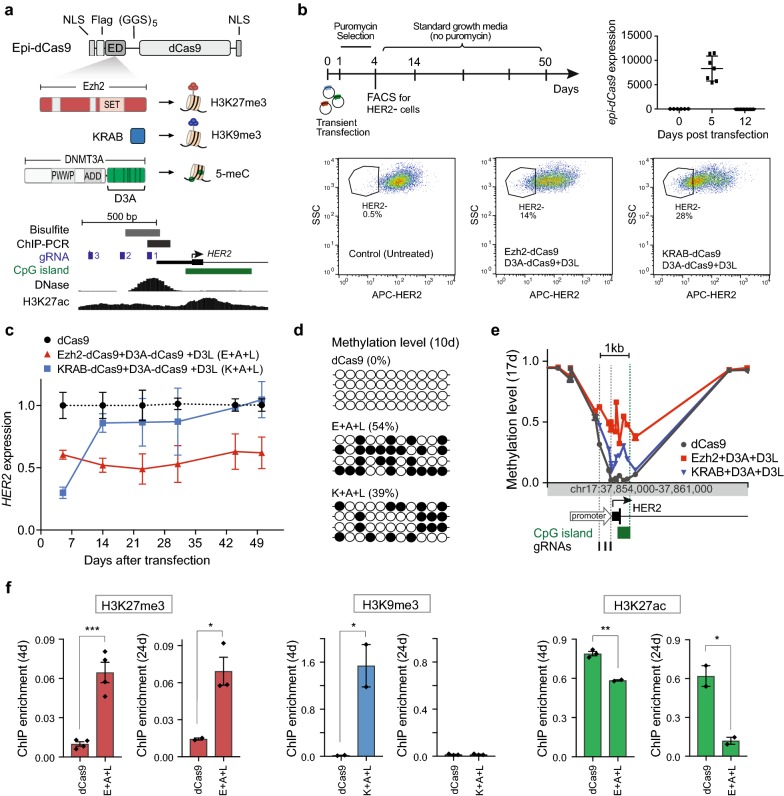
Hit-and-run epigenetic editing by Ezh2-dCas9, but not KRAB-dCas9, with targeted DNA methylation establishes and maintains a local heterochromatic environment. **a** Schematic representation of epi-dCas9. dCas9 fusions to effector domains (ED) contained N-terminal and C-terminal nuclear localization domains (NLSs), as well as an N-terminal 3XFLAG epitope tag with a 15-aa linker [(GGS)_5_] separating dCas9 and the EDs. Effector domains KRAB, Ezh2 and DNMT3A with domains are shown next to epigenetic marks they induce. A detailed view around the human *HER2* promoter region shows an annotated CpG island (green bar), gRNAs targeting HER2 promoter region (blue boxes) and the regions interrogated by bisulfite cloning (gray bar) and ChIP-qPCR (black bar). The transcription start site (TSS) is indicated. UCSC browser tracks of DNAse1 hypersensitivity and H3K27ac ChIP-seq enrichment at the *HER2* locus (human genome assembly hg19) are also shown. **b** Diagram of experimental design to assay for long-term repression using flow cytometry to select for low *HER2* expression (HER2−) 4 days after transient transfection assays. Relative epi-dCas9 expression was determined by RT-qPCR in HCT116 cells after co-transfection of plasmids expressing dCas9,KRAB-dCas9 and Ezh2-dCas9 with three gRNAs targeted to the *HER2* promoter (Dunnett’s test *P* < 0.001; *n *= 3 independent experiments per epi-dCas9 fusion; mean ± SEM). Representative flow cytometry data for *HER2* expression in HCT116 cells treated with plasmids expressing Ezh2 + D3A + D3L (Ezh2-dCas9, D3A-dCas9 and overexpressed D3L) or KRAB + D3A + D3L (KRAB-dCas9, D3A-dCas9 and overexpressed D3L) in the presence of three gRNAs targeting the *HER2* promoter. HER2− cells collected are indicated. Untreated control to identify HER2+ population is shown on the left. **c** Long-term repression of *HER2* mRNA levels in HER2− cells was monitored for 50 days using RT-qPCR after co-transfection of plasmids expressing the indicated epi-dCas9 fusions and three gRNAs targeted to the *HER2* promoter (*n *= 3 independent experiments each; mean ± SEM). **d** Average methylation levels were determined by bisulfite cloning at the *HER2* promoter 10 days after transfection with the indicated Ezh2-dCas9 and KRAB-dCas9 cocktails or dCas9 (no effector domain). Each line represents a single clone, filled and empty circles represent a single methylated or unmethylated CpG, respectively. Average % methylation is indicated for each treatment. **e** Methylation level of CpG probes along 7 kb of the *HER2* locus 17 days after HCT116 cells were co-transfected with indicated epi-dCas9 and three gRNAs targeting the *HER2* promoter. CpG Island is shown in green. **f** Transient (4 days) and persistent (24 days) histone methylation was determined by ChIP-qPCR at the *HER2* promoter in HCT116 cells co-transfected with either dCas9 with no ED or the indicated epi-dCas9 treatment cocktail and three gRNAs targeted to the *HER2* promoter (Student’s *t* test, *P < 0.05, ***P < 0.001; *n *= 2–4; mean ± SEM). Due to higher cell number required for ChIP-qPCR, cells were cross-linked immediately after puromycin selection (4 days); sort for HER2− cells by FACS was omitted. H3K27me3 and H3K9me3 ChIP enrichment was assayed for Ezh2 + D3A + D3L and KRAB + D3A + D3L, respectively and compared to dCas9 with no ED. Decrease of H3K27ac enrichment was measured by ChIP-qPCR at the *HER2* promoter in HCT116 at 4 and 24 days after co-transfection with three gRNAs targeted to the *HER2* promoter and dCas9 with no ED or the epi-dCas9 treatment cocktail Ezh2 + D3A + D3L (Student’s *t* test, **P < 0.01, *P < 0.05; *n *= 2–3; mean ± SEM)

We have previously created a toolbox of dCas9 fusions with epigenetic effector domains (epi-dCas9), which can deposit their respective epigenetic marks at the endogenous *HER2* target site in HCT116 cells when transfected individually [26]. DNMT3A-dCas9 (D3A-dCas9) establishes 5C DNA methylation, Ezh2[FL]-dCas9 (Ezh2-dCas9) establishes H3K27 trimethylation, and KRAB-dCas9 induces H3K9 trimethylation at the target site (Fig. 1a). Individual epi-dCas9 fusions were capable of transient *HER2* repression, but they failed to induce long-term repression. Although reports have emerged that a combination of the KRAB repressor domain and DNA methyltransferases is required to engineer epigenetic memory [25, 27], we were unable to use this combination to engineer long-term repression at the *HER2* locus in HCT116 cells [26]. In this study, we aim to gain a better understanding of the requirements to stably engineer epigenetic memory at different loci. First, we compared combinatorial treatment of DNA methylation with either KRAB or with the histone methyltransferase Ezh2 and the effect on long-term repression and establishment and maintenance of a repressive epigenetic state. For this purpose, HCT116 cells were co-transfected with either Ezh2-dCas9 or KRAB-dCas9 cocktail, plus D3A-dCas9 and D3L, and three gRNAs targeting the *HER2* gene promoter (Fig. 1a). To more easily monitor gene expression over time, we took advantage of the transmembrane properties of the HER2 protein and employed flow cytometry to collect cells with no/low APC-HER2 fluorescence (HER2−) 4 days after transfection (Fig. 1b). On average, 14% of the cells from three biological replicates were HER2− after exposure to the Ezh2-dCas9 cocktail and 28% were HER2− after treatment with the KRAB cocktail (Fig. 1b). Thus, the stronger transient repressive activity of KRAB-dCas9 was reflected in the higher number of HER2− cells, indicating that this method can be used to monitor *HER2* gene expression (repression of *HER2* expression also leads to persistent reduction in HER2 protein; see Additional file 3: Figure S2A). HER2− cells were then maintained without selection for 50 days after transfection. Unsorted cells transfected with dCas9 (no effector domain) were grown in parallel and used as a control. Time points for individual experiments were determined by expansion of sorted cells to 80% confluency (see "Materials and methods"). As expected, transfected epi-dCas9 fusions were transiently expressed at high levels 5 days after transfection, but expression of dCas9 fusions was no longer detectable 12 days after transfection (Fig. 1b). Ezh2-dCas9 in combination with D3A-dCas9 and D3L produced persistent twofold repression for the entire 50-day period, supporting the hypothesis that a repressive state was established and maintained through approximately 57 mitotic cell divisions (doubling time ~ 21 h) (Fig. 1c). Although KRAB-dCas9 together with D3A-dCas9 and D3L initially repressed *HER2* expression by 3.3-fold, repression was lost and normal *HER2* expression was re-established by day 14 after transfection. Although we sorted and expanded HER2− cells, we observed persistent repression of only twofold. This could either represent partial repression in individual cells or result from a bimodal population where a subset of cells maintained the HER2− state and another subset regained *HER2* expression. We performed FACS analysis to evaluate the HER2 protein level in cells 54 days post-transfection (50 days after the initial sort). FACS analysis demonstrates all-or-none maintenance of silencing events (Additional file 3: Figure S2B). We observed 31% HER2− cells in the population treated with Ezh2-dCas9, D3A-dCas9 and D3L, while 2% of cells remained HER2− after exposure to the KRAB-dCas9 cocktail. No HER2− cells were present after treatment with dCas9 (no effector).

We then investigated whether the persistent change in *HER2* expression was accompanied by a change in the chromatin environment at the *HER2* promoter. We interrogated a 229-bp region in the *HER2* promoter (Fig. 1a) by bisulfite sequencing to determine the methylation status in HER2− cells 10 days after treatment with Ezh2-dCas9 or KRAB-dCas9 cocktail (plus D3A-dCas9 and D3L) and three gRNAs targeting the *HER2* gene promoter. Treatment with KRAB-dCas9 cocktail resulted in 39% methylation, while Ezh2-dCas9 facilitated treatment showed increased methylation of 54% (Fig. 1d). No methylation was observed in cells treated with dCas9 with no effector domain (Fig. 1d). We further wanted to investigate whether hypermethylation was associated with spreading of DNA methylation. We used the Illumina Infinium HumanMethylationEPIC (EPIC) array to investigate DNA methylation beyond the target site 17 days after treatment. Cells treated with Ezh2-dCas9/D3A-dCas9 and D3L showed hypermethylation of all 12 CpGs in the *HER2* promoter region and the promoter-proximal 5′UTR region with an average methylation of 48% as compared to 7% in dCas9 control samples (Fig. 1e). In addition to the 416-bp nucleation site (spanned by the 3 *HER2* gRNAs), hypermethylation spread through the downstream CpG island, creating a 1.25-kb hypermethylated region (Fig. 1e). On the other hand, only a single *HER2* promoter CpG was hypermethylated 17 days after treatment with KRAB-dCas9/D3A-dCas9 and D3L. The single hypermethylated CpG was located in the CpG island downstream of the gRNA target region and was not associated with *HER2* gene repression (Fig. 1c). This suggests that higher initial methylation level (Fig. 1d) and/or spreading of DNA methylation (Fig. 1e) facilitated by Ezh2-dCas9 plus DNA methyltransferases enables persistence of a repressive chromatin state and thereby enables long-term epigenetic memory at the target locus.

We next determined whether co-targeting with Ezh2-dCas9 or KRAB-dCas9 resulted in deposition and maintenance of the corresponding histone marks (H3K27me3 and H3K9me3, respectively). Because cell number was limited after sorting for HER2− cells, we performed ChIP-qPCR on unsorted cells for the transient time point (4 days), but used the collected HER2− population (Fig. 1b) for the persistent time point (24 days). ChIP-qPCR confirmed the presence of the expected histone marks 4 days after transfection (Fig. 1f), when epi-dCas9 was still present (see Fig. 1b). In addition, the 6.6-fold increase in H3K27 trimethylation at the *HER2* promoter mediated by Ezh2-dCas9 was maintained at a similar level (4.7-fold) at 24 days after transfection (Fig. 1f). On the other hand, KRAB-dCas9 initially triggered a significant increase in H3K9 trimethylation (102-fold), but this epigenetic mark was completely lost 24 days after transfection; this loss corresponded to the loss of *HER2* repression. To determine whether the gain in the repressive H3K27 methylation mark is accompanied by a loss of the active H3K27 acetylation mark, we performed H3K27ac ChIP-qPCR. Indeed, we observed a 1.4-fold decrease in H3K27ac 4 days after transfection, which was more pronounced (5.2-fold) 24 days after exposure to Ezh2-dCas9 plus D3A-dCas9 and D3L (Fig. 1f). These data clearly demonstrate that targeting Ezh2-dCas9, D3A-dCas9 and D3L establishes and maintains a repressive chromatin environment comprised of DNA and histone methylation.

### Long-term silencing by combinatorial treatment with Ezh2-dCas9 or KRAB-dCas9 is locus specific

**Fig. 2:**
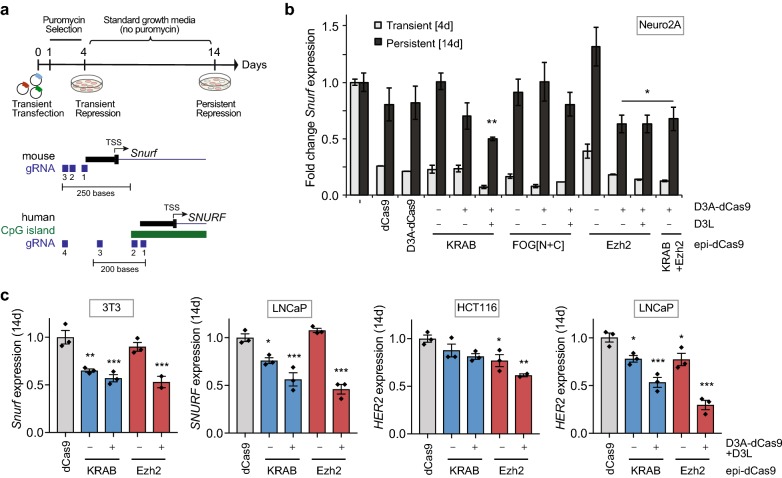
Establishing long-term repression by combinatorial Ezh2-dCas9 or KRAB-dCas9 treatment is context-dependent. **a** Diagram of experimental design for transient transfection assays with puromycin enrichment to assay for transient (4 days) and long-term (14 days) repression. A detailed view around the mouse *Snurf* and human *SNURF* promoter region shows gRNAs target sites (blue boxes) and an annotated CpG island (green bar). The transcription start site (TSS) is indicated. **b** RT-qPCR of endogenous *Snurf* mRNA levels in Neuro2A cells 4 days (transient) and 14 days (long-term) after co-transfection of plasmids expressing three gRNAs targeted to the *Snurf* promoter and the indicated epi-dCas9 fusions individually or in combination with D3A-dCas9 with or without simultaneous overexpression of DNMT3L (D3L). *Snurf* mRNA levels were compared to dCas9 with no ED (Tukey-test, *P < 0.05, **P < 0.01, *n *= 2–3; mean ± SEM). **c** Long-term repression (14 days) resulting from epi-dCas9 fusions ± targeted DNA methylation (D3A-dCas9 and overexpressed D3L) compared to dCas9 with no ED was determined at two different target genes (*Snurf, SNURF or HER2*) by RT-qPCR in the cell lines indicated (Dunnett’s test, *P < 0.05, **P < 0.01, **P < 0.001; *n *= 3; mean ± SEM)

Co-targeting DNA methylation with Ezh2-dCas9, but not with KRAB-dCas9, stably silenced *HER2* expression. We then wanted to investigate whether another locus that is readily silenced by a combination of KRAB-dCas9 with D3A-dCas9 and D3L also responds to combinatorial Ezh2-dCas9 treatment, or whether the two modes of action are mutually exclusive. For this purpose, we evaluated different combinations of epi-dCas9 fusions using three gRNAs targeting the *Snurf* promoter in Neuro2A cells. Transient repression was measured 4 days post-transfection under puromycin selection to enrich for transfected cells, while persistent repression was measured after cells were grown for an additional 10 days in puromycin-free media (Fig. 2a). Informed by our results (Fig. 1c) and previous reports [25, 26], we evaluated persistent silencing 14 days after transfection. In addition to Ezh2-dCas9, we included dCas9-FOG [N + C], which deposits similar levels of H3K27 trimethylation when targeted to the *HER2* promoter [26]. All epi-dCas9 fusions were able to repress *Snurf* gene expression temporarily, ranging from 2.6- to 14.2-fold downregulation (Fig. 2b). Importantly, dCas9 without any effector domain was also able to repress *Snurf* gene expression 3.6-fold. This effect, also known as CRISPR inhibition (or CRISPRi), is due to binding of dCas9 near the transcription start site and subsequent blocking access of endogenous factors required for transcription [40]. As expected, individual epi-dCas9 fusions (KRAB-dCas9, Ezh2-dCas9, D3A-dCas9, dCas9-FOG [N + C]) could cause transient repression, but in each case *Snurf* gene expression was re-established by 14 days after transfection. The observed CRISPRi effect of dCas9 without an effector domain was also of transient nature. Long-term repression of the *Snurf* gene by KRAB-dCas9 required co-expression of both D3A-dCas9 and D3L (twofold; Fig. 2b). On the other hand, long-term repression of the *Snurf* gene by Ezh2-dCas9 was observed in combination with D3A-dCas9 (1.6-fold) or after combinatorial treatment with D3A-dCas9 and D3L (1.6-fold). dCas9-FOG [N + C] by itself, or in combination with D3A-dCas9 and D3L, was not able to confer long-term repression of the *Snurf* gene. Similar results were obtained when targeting the *TRPM4* promoter in C42B cells (Additional file 4: Figure S3). Interestingly, these data demonstrate that targeted H3K27me3 deposition by dCas9-FOG [N + C] is not sufficient for long-term repression, even in the presence of targeted DNA methyltransferase, while H3K27me3 deposition by Ezh2-dCas9, in combination with targeted DNA methylation by D3A-dCas9 and overexpression of D3L, does lead to long-term repression of *Snurf* expression. This suggests that either interaction of the epigenetic effectors themselves (Ezh2 and D3A) or recruitment of PRC2 complex partners specific to Ezh2 is necessary for long-term repression.

We next investigated whether simultaneous recruitment of the histone methyltransferase Ezh2 and DNA methyltransferases is also required in other cell types. It has previously been shown that long-term repression by a targetable fusion with the KRAB repressor domain required simultaneous recruitment of the histone methyltransferase DNMT3A and DNMT3L in some, but not all cell types tested [25]. We observed that KRAB-dCas9 by itself was able to induce modest long-term repression of *Snurf* in 3T3 cells as well as *SNURF* and *HER2* in LNCaP cells. However, long-term silencing efficiency was increased when a combination of KRAB-dCas9, D3A-dCas9 and D3L was used (Fig. 2c). Efficient long-term repression by Ezh2-dCas9 also required addition of D3A-dCas9 and D3L. We note that the combination of D3A-dCas9 and D3L with Ezh2-dCas9 or KRAB-dCas9 induced comparable levels of long-term repression: Ezh2-dCas9 facilitated 1.9- and 2.2-fold repression, while KRAB-dCas9 established 1.8- and 1.7-fold repression of *Snurf/SNURF* expression in 3T3 and LNCaP cells, respectively. In LNCaP cells, addition of D3A-dCas9 and D3L to Ezh2-dCas9 or KRAB-dCas9 established 3.4- and 1.9-fold persistent *HER2* repression, respectively. Among the two loci tested in different mouse and human cell types, only the *HER2* locus in HCT116 cells was resistant to long-term silencing by KRAB-dCas9 in combination with D3A-dCas9 and D3L. This demonstrates that different combinations of targeted histone and DNA methyltransferases are required to achieve maximal repression at the same locus in different cell types or at different loci in the same cell type. Altogether, these data indicate that there are locus- and/or cell-type-specific requirements for the specific tools needed to silence an endogenous gene by targeted recruitment of histone and DNA methylases.

### Genome-wide H3K27ac analysis demonstrates that combinatorial Ezh2-dCas9 treatment leads to loss of H3K27ac specific to the *HER2* target promoter

**Fig. 3:**
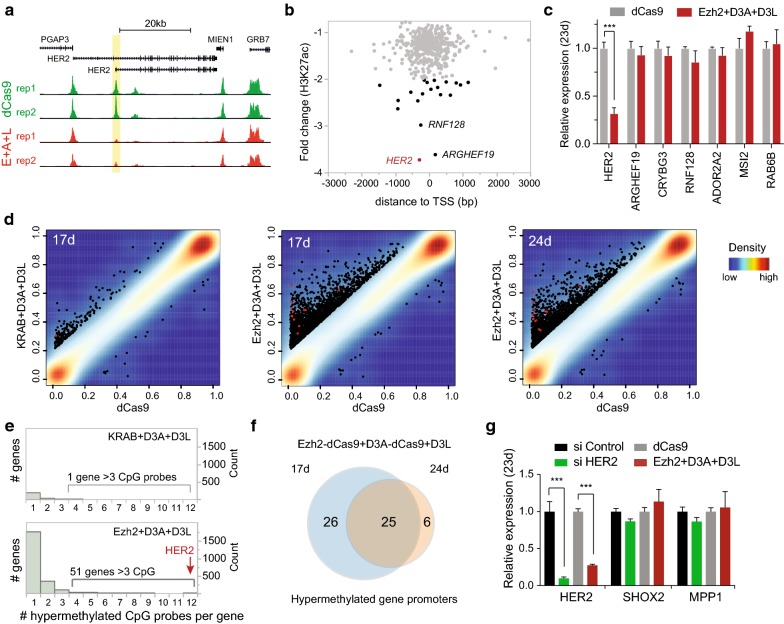
Combinatorial treatment with Ezh2-dCas9, D3A-dCas9 and D3L establishes persistent DNA and histone methylation specifically at the *HER2* target locus. **a** UCSC browser snapshot of H3K27ac ChIP-seq enrichment at the *HER2* locus (human genome assembly hg19). Browser tracks are shown for biological replicates 24 days after co-transfection with indicated epi-dCas9 cocktail and 3 gRNAs targeting the *HER2* promoter (dCas9 control in top two panels, Ezh2 + D3A + D3L treated cells in bottom two panels). Targeted *HER2* promoter region containing H3K27ac peak is highlighted in yellow. RefSeq genes are indicated. **b** Scatterplot of the fold change of H3K27ac binding (-dCas9/Ezh2 + 3A + 3L) after co-targeting of Ezh2 + D3A + D3L (24 days) against peak center distance from the transcription start site (TSS). 469 H3K27ac binding peaks that are specific to the dCas9 control were plotted. Black dots represent gene promoters with a more than twofold reduction of H3K27ac binding. Reduction of H3K27ac binding at the *HER2* promoter is indicated with a red dot. **c** RT-qPCR at six loci with reduced H3K27ac ChIP binding 23 days after treatment with Ezh2-dCas9/D3A-dCas9 plus D3L. Expression levels were calculated relative to GAPDH (Sidak’s multiple comparisons test, ***P < 0.001, *n *= 3; mean ± SEM). **d** Distribution of DNA methylation levels in promoter regions. Average DNA methylation levels of HCT116 cells co-transfected with plasmids expressing three gRNAs targeting the *HER2* promoter, and epi-dCas9 cocktails expressing D3A-dCas9 and D3L with either KRAB-dCas9 (KRAB + D3A + D3L) or Ezh2-dCas9 (Ezh2 + D3A + D3L) are plotted against control cells transfected with same gRNAs and dCas9 with no ED. Hypermethylated *HER2* CpG probes in promoter regions are colored in red, all other differentially methylated promoter CpG probes are colored in black. Promoter regions encompass 1500 bp upstream of the TSS as well as the 5′UTR. **e** Frequency of hypermethylated CpG probes per gene. Gene promoters containing more than three hypermethylated probes are considered differentially methylated promoters. After treatment with Ezh2 + D3A + D3L the *HER2* target promoter shows strongest differential methylation represented by 12 hypermethylated promoter CpGs (red arrow). **f** Persistent hypermethylation of 25 gene promoters. Venn diagram depicts hypermethylated gene promoters with > 3 hypermethylated promoter probes 17 and 24 days after targeting the *HER2* promoter with Ezh2-dCas9 + D3A-dCas9 + D3L. **g** RT-qPCR at potential off-target sites 23 days after treatment with indicated epi-dCas9. As a control, *HER2* expression was knocked down by *HER2* siRNA and compared to siRNA control treatment. Expression levels were calculated relative to GAPDH (Tuckey’s test, ***P < 0.001; *n *= 3; mean ± SEM)

We demonstrated that the targeted increase in the repressive histone mark H3K27me3 is associated with long-term loss of H3K27ac (Fig. 1f), which is associated with distal regulatory regions and with gene promoters [9]. Therefore, loss of H3K27ac in promoter regions can be used as an indicator for reduced expression. We performed ChIP-seq 24 days after transfection with either dCas9 (control) or Ezh2-dCas9 cocktail (including D3A-dCas9 and D3L) to analyze H3K27ac signals genome-wide; comparisons of H3K27ac peaks showed strong correlation between biological replicates (Spearman’s *ρ* > 0.98; Additional file 5: Figure S4). Biological replicates were then combined, and the top 15,000 robust ChIP-seq peaks were called in control and combinatorial Ezh2-dCas9 treatment, independently (Additional file 6: Table S2). When we compared the robust peaks, 13,528 (91%) of H3K27ac peaks found in the control treatment were also present in cells that received Ezh2-dCas9 in combination with D3A-dCas9 and D3L (Additional file 7: Figure S5A and S5B). As expected, a H3K27ac binding peak at the *HER2* target site was only identified in control cells, but no robust binding signal was observed after combinatorial Ezh2-dCas9 treatment (Fig. 3a). We were interested whether other promoters showed similarly reduced levels of H3K27ac, which may indicate loss of transcription of the associated gene. 1392 H3K27ac peaks were only present in control cells, but not in cells treated with Ezh2-dCas9 cocktail. Among these control-specific peaks, only 469 H3K27ac binding peaks were localized within ± 1 kb of the transcription start site (TSS). H3K27ac binding enrichment at these 469 TSS proximal regions was calculated after normalization using DiffBindR program [37], and the change in H3K27ac binding signals between control and combinatorial Ezh2-dCas9 treatment was calculated. Only 20 of the 469 promoter-proximal regions showed a more than twofold reduction in H3K27ac binding (Fig. 3b, Additional file 6: Table S2). The strongest reduction, which was 3.7-fold, was observed at the *HER2* target site with the H3K27ac peak centered in the proximal promoter (− 288 bp relative to TSS). *ARHGEF19* and *RNF128* showed a reduction of 3.6- and threefold, respectively. The H3K27ac ChIP-seq signal was smaller at *ARHGEF19* and *RNF128* (approximately half and a fourth, respectively) than at the *HER2* target promoter. It is noteworthy that among the 20 promoter binding peaks showing more than twofold reduction, H3K27ac ChIP-seq signal was highest at the *HER2* promoter (Additional file 7: Figure S5C). If H3K27ac changes were due to off-target binding of epi-dCas9 fusions, one would expect an overlap with predicted Cas9 off-targets. We used Cas-OFFinder [41] to identify predicted Cas9 off-targets in the human genome with a ‘NGG’ PAM sequence and allowing up to three mismatches to each of the three *HER2* gRNA target sequences. There were 3, 58 and 28 predicted off-target sites for *HER2* gRNAs 1,2 and 3, respectively (Additional file 2: Table S1). Using bedtools, we determined that there was no overlap between predicted off-target sites and the 469 TSS proximal regions that showed reduced H3K27ac binding. To determine whether changes in H3K27ac binding affect gene expression, we performed RT-qPCR of the top 10 promoters with reduced H3K27ac enrichment. We found that 4 out of the 10 genes (*GBX1, ADCK5, BRINP3, CD8A*) were either not expressed or not detectable by RT-qPCR in HCT116 cells. The remaining six gene promoters did not show a change in gene expression 23 days after transfection (Fig. 3c). Thus, we observed very few off-target H3K27ac changes in the genome of the transfected cells and did not observe any changes in gene expression at the off-target loci tested.

### Global analysis of persistent DNA methylation after combinatorial targeting with Ezh2-dCas9 or KRAB-dCas9 and DNA methyltransferase (D3A-dCas9 and D3L)

We have demonstrated that treatment with Ezh2-dCas9, plus D3A-dCas9 and DNMT3L, cannot only establish, but also stably maintain DNA methylation (Fig. 1d, e). Some groups have reported very precise targeted methylation [25, 42, 43], and others have found significant amounts of off-target methylation [43–45]. We used the Illumina Infinium HumanMethylationEPIC (EPIC) array to obtain a global view of DNA methylation at human promoters. Global DNA methylation was analyzed 17 days after cells were treated with *HER2* gRNAs and Ezh2-dCas9 or KRAB-dCas9 cocktails (containing D3A-dCas9 + D3L) and compared to cells transfected with dCas9 (no effector domain); biological replicates showed strong bivariate correlation (*R*^2^ > 0.99; Additional file 8: Figure S6). Methylation levels are recorded as beta values (*β*), which are determined by the ratio of intensities between methylated (*M*) and unmethylated (*U*) alleles (*β* = *M*/*M* + *U*). EPIC arrays reliably detect a change in beta values of 0.2 (methylation change of 20%) with FDR < 0.01. Since hypermethylation of gene promoters is frequently associated with transcriptional repression, average methylation was calculated at promoter regions encompassing from 1.5 kb upstream of the transcription start site (TSS) through the 5′ UTR. On average, each promoter region is represented by 14 CpG probes on the EPIC array. Promoter CpGs showing a 20% increase in DNA methylation are highlighted in Fig. 3d regardless of the original promoter methylation state. Differentially methylated regions (DMRs) usually involve adjacent CpGs or a group of CpGs close together that have different methylation patterns between samples, with a minimum requirement of > 3 differentially methylated CpGs [46, 47]. We determined the frequency of methylated CpGs occurring at each promoter region and observed that the majority of promoter regions only contained a single hypermethylated CpG after transfection with the epi-dCas9 fusions (Fig. 3e). For example, KRAB-dCas9 in combination with D3A-dCas9 and D3L resulted in hypermethylation of 223 CpGs corresponding to 199 genes (GenCode Basic v12). 91% of these genes contained only a single hypermethylated CpG in the entire promoter region (Additional file 9: Table S3). Only one possible off-target gene with > 3 hypermethylated promoter CpGs was identified. The *SHOX2* gene promoter contains 4 hypermethylated CpGs with an average methylation of 33% as compared to 6% in dCas9 control cells. We note that the KRAB-dCas9 plus D3A-dCas9 and D3L treated cells were only transiently exposed to DNA methyltransferase. Our results demonstrate that hit-and-run DNA methyltransferase targeting does not display promiscuous off-target activity under these experimental conditions.

Ezh2-dCas9 treatment in combination with D3A-dCas9 and D3L resulted in hypermethylation of more CpG probes (2966) than the KRAB-dCas9 experiment. However, even in this case, only 51 promoters contained > 3 hypermethylated CpGs (Additional file 9: Table S3). Importantly, the targeted *HER2* promoter contained the most hypermethylated CpGs (12 hypermethylated CpGs), showing an average methylation of 48% as compared to 7% in dCas9 control samples.

It has previously been reported that gain of off-target methylation by transient overexpression of DNMT3A-dCas9 fusions is of a transient nature [45, 48]. We wanted to determine whether promoter hypermethylation was stabilized 17 days after combinatorial Ezh2-dCas9 treatment with D3A-dCas9/D3L. DNA methylation 24 days after transfection showed high concordance (93%) with 1876 overlapping hypermethylated CpGs at 17 and 24 days after transfection with 2966 and 2018 hypermethylated CpGs, respectively. We then compared the 89 off-target sites predicted by Cas-OFFinder (as described earlier; Additional file 2: Table S1) to the 1876 overlapping hypermethylated CpGs. It is possible that a hypermethylated CpG is not directly within, but adjacent to a predicted off-target site. We therefore expanded the hypermethylated region by the length of a nucleosome up- and downstream of the CpG (± 146 base pairs). There were no overlaps between predicted off-target sites and the expanded hypermethylated regions. Using the criteria of > 3 hypermethylated CpGs per gene promoter, half of the gene promoters we identified to be hypermethylated at 17 days post-transfection were still hypermethylated at 24 day post-transfection (Fig. 3f; Additional file 9: Table S3). Among the 25 persistently hypermethylated gene promoters (represented by 132 CpG probes), *HER2* still ranked highest having 12 hypermethylated CpGs and an average methylation level of 46%. *SHOX2,* which was the only potential off-target identified by D3A-dCas9/D3L treatment with KRAB-dCas9, was also among the 25 persistently hypermethylated gene promoters. To evaluate the impact of DNA methylation on expression of potential off-target sites, we performed RT-qPCR in cells 23 days after co-transfection with the Ezh2-dCas9 cocktail (plus D3A-dCas9/D3L) and compared expression to control cells treated with dCas9 without effector domain (Fig. 3g). To evaluate possible downstream effects of *HER2* repression, HCT116 cells with siRNA mediated knockdown of HER2 were used as a control. *SHOX2* and *MPP1* encompassed 9 and 4 hypermethylated CpG probes, respectively. Neither *SHOX2* nor *MPP1* showed changes in gene expression when compared to control samples, while *HER2* was robustly downregulated in cells treated with HER2 siRNA and in cells 23 days after treatment with Ezh2-dCas9 cocktail (Fig. 3g).

We further hypothesized that true off-targets from combinatorial Ezh2-dCas9/DNMT3A-dCas9 and D3L treatment would contain both an increase in DNA methylation and a decrease in H3K27 acetylation. When we overlapped the 25 hypermethylated CpG probes identified at 24 days after transfection with H3K27ac ChIP peaks that showed reduced binding in Ezh2-dCas9/D3A-dCas9/D3L treated cells, only the target gene *HER2* was identified. Taken together, our data demonstrate that the Ezh2-dCas9 cocktail can engineer epigenetic memory at the *HER2* locus conferring long-term repression with very few potential off-targets. Persistent repression is associated with a stably maintained epigenetic switch to a heterochromatin environment stably marked by both, histone H3K27 trimethylation and DNA methylation.

### Alternative strategies for combinatorial epi-dCas9 recruitment while maintaining a reduced number of gRNAs

Global ChIP-seq and DNA methylation analysis revealed very few potential off-target sites. However, the use of multiple gRNAs constitutes an increased risk for off-target binding of epi-dCas9 fusions. Since a combination of different epi-dCas9 fusions is required to engineer persistent epigenetic silencing, we explored alternative recruitment strategies that will allow us to recruit multiple epi-dCas9 fusions while maintaining a reduced number of gRNAs. First, we explored whether a single gRNA could facilitate targeted epigenetic silencing with multiple epi-dCas9 fusions. Multiple epi-dCas9 fusions are exploiting the same gRNA, possibly leading to DNA binding competition. To test this, we used two epi-dCas9 fusions (KRAB-dCas9 and D3A-dCas9) in combination with D3L, with a pool of three gRNAs or a single gRNA targeting the *Snurf* promoter in Neuro2A cells. Surprisingly, we found that a single gRNA was just as effective as the gRNA pool in inducing long-term repression. *Snurf* gRNA 1 induced 2.3-fold repression, while the gRNA pool (gRNAs 1,2 and 3) resulted in 1.6-fold repression of *Snurf* expression (Additional file 10: Figure S7A).

Alternatively, recruitment of effector domains through the gRNA component may offer a solution to increasing the number of epi-dCas9 fusions while reducing the number of gRNAs and therefore the number of possible off-target sites (Additional file 10: Figure S7B). In previous studies, gRNA scaffolds were used that contain two MS2 aptamers (MS2-gRNA), one in the gRNA tetraloop and one in stem loop 2. These aptamers can be recognized by the MS2 bacteriophage coat protein (MCP), to which various effector domains (VP64, P65, P65-HSF1, SS18 or HP1) can be attached [33, 49–52]. For our study, we first created fusions of KRAB and Ezh2 to either one or a tandem array of two MCPs, resulting in either 1x or 2x MCP-effector fusions. MS2-gRNAs were designed to target the same endogenous sites as their respective traditional gRNAs. When dCas9 was targeted to the *HER2* promoter by a pool of three MS2-gRNAs, 2xMCP-KRAB showed twofold repression at 4 days post-transfection in HCT116 cells, while 1xMCP-KRAB was not able to repress *HER2* expression. We then determined whether effector pairing of epi-dCas9 and MCP fusions could increase the repressive capacity of gRNA-based recruitment. Simultaneous recruitment of KRAB-dCas9 and 2xMCP-KRAB decreased repression to 1.3-fold, perhaps by destabilizing the dCas9 complex. However, the direct fusion of KRAB to the N-terminus of dCas9 (KRAB-dCas9) was most effective resulting in tenfold repression of *HER2* expression. 1xMCP-Ezh2 and 2xMCP-Ezh2 fusions were unable to repress *HER2* gene expression. We additionally performed this test at the *Snurf* promoter in Neuro2A cells. Again, only the direct fusion of KRAB-dCas9 was able to persistently repress *Snurf* expression, while one or two MS2-gRNAs recruiting 2xMCP-KRAB to the dCas9 complex did not cause significant long-term repression (Additional file 10: Figure S7B). While the MS2-gRNA recruitment strategy holds great potential, direct epi-dCas9 fusions in combination with a single-gRNA induced persistent epigenetic silencing more efficiently under these conditions.

### Co-targeting of full-length DNMT3L is necessary and sufficient to enable persistent gene silencing by Ezh2-dCas9

**Fig. 4:**
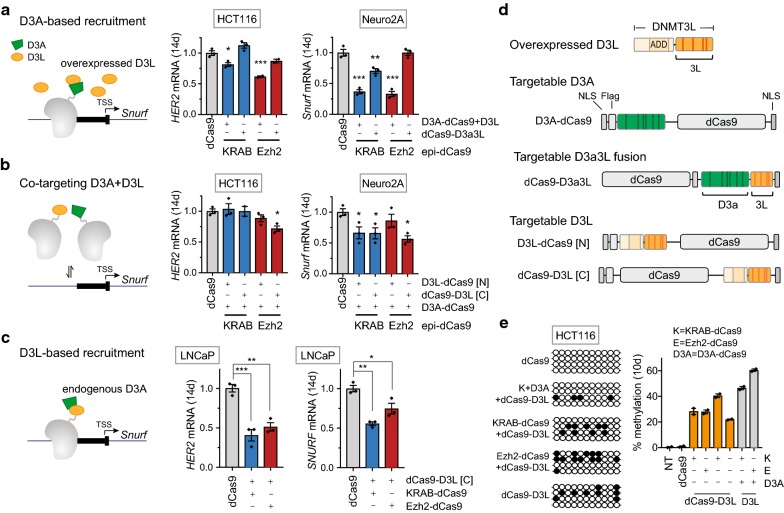
Full-length DNMT3L is required and sufficient to establish long-term repression facilitated by KRAB-dCas9 and Ezh2-dCas9. Long-term repression was evaluated using three different approaches for targeted DNA methylation: **a** D3A-based recruitment (D3A-dCas9 + overexpressed D3L), **b** Co-targeting of D3A + D3L (D3A-dCas9 + dCas9-D3L) and **c** D3L-based recruitment (dCas9-D3L). **a** Comparison of the D3A-based recruitment approach with the targetable D3a3L fusion (dCas9-D3a3L), *HER2* mRNA levels in HCT116 cells and *Snurf* mRNA levels in Neuro2A cells were determined by RT-qPCR 14 days after co-transfection of plasmids expressing the indicated epi-dCas9 fusions with the three gRNAs targeted to the *HER2* or *Snurf* promoter (Dunnett’s test, *P < 0.05, **P < 0.01, ***P < 0.001; *n *= 3; mean ± SEM). **b** Long-term repression by co-targeting D3A and D3L fusions. Performance of D3L fusions to the N or C terminus of dCas9 was compared. *HER2* mRNA levels in HCT116 cells and *Snurf* mRNA levels in Neuro2A cells were determined by RT-qPCR 14 days after co-transfection of plasmids expressing the indicated epi-dCas9 fusions with the three gRNAs targeted to the *Snurf* or *HER2* promoter (Dunnett’s test, *P < 0.05, **P < 0.01, ***P < 0.001; *n *= 3; mean ± SEM). **c** Long-term repression was evaluated by RT-qPCR 14 days after co-transfection of dCas9-DNMT3L with KRAB-dCas9 or Ezh2-dCas9 with gRNAs targeted to the *SNURF* and *HER2* promoter in LNCaP cells. Expression levels were compared to dCas9 with no ED (Dunnett’s test, *P < 0.05, **P < 0.01, ***P < 0.001; *n* = 3; mean ± SEM). **d** Schematic of overexpressed full-length DNMT3L and design of dCas9 fusions. DNMT3A or DNMT3L were fused to the N-terminus of dCas9 (D3A-dCas9 and D3L-dCas9, respectively) or the C terminus of dCas9 (dCas9-D3L). dCas9-D3a3L contains D3a and the C-terminal portion of D3L (D3a3L) fused to the N-terminus of dCas9. **e** Average methylation levels were determined by bisulfite cloning (left panel) and high-throughput bisulfite amplicon sequencing (right panel) at the *HER2* promoter 10 days after transfection with the indicated Ezh2-dCas9 and KRAB-dCas9 cocktails or dCas9 (no effector domain). Methylation after treatment with only dCas9-D3L and three *HER2* gRNAs was also evaluated. Bisulfite cloning results are displayed as lollipop plots. Each line represents a single clone, filled and empty circles represent a single methylated or unmethylated CpG, respectively. Analysis of bisulfite amplicons sequencing was performed from two biological replicates. Average % methylation is plotted for each indicated epi-dCas9 treatment. Methylation obtained with targetable dCas9-D3L (orange bars) was compared with methylation status after combinatorial KRAB-dCas9 (K) and Ezh2-dCas9 (E) with D3A-dCas9 (D3A) and overexpressed D3L (gray bars). All treatments resulted in significant *HER2* target methylation when compared to cells treated with dCas9 or cells that were not transfetcted (NT) (Dunnett’s test, ***P < 0.001; *n* = 2; mean ± SEM)

The catalytic activity of the de novo DNA methyl transferase DNMT3A is enhanced by dimerization with DNMT3L [28]. In fact, a direct fusion of the C-terminal portion of DNMT3L to the catalytic domain of Dnmt3a (D3a3L) to a DNA targeting module (zinc finger protein or dCas9) enabled efficient DNA methylation at endogenous target sites [29, 42, 53]. We have shown that the D3A-based recruitment approach can initiate long-term repression in the presence of overexpressed DNMT3L (Fig. 4a). Alternatively, we sought to explore the impact of a targetable version of DNMT3L on long-term silencing by co-targeting D3A-dCas9 and targetable DNMT3L (D3L) fusions (Fig. 4b). In order to avoid overexpression of the catalytically active DNMT3A, we tested the ability of targetable D3L fusions to recruit endogenous D3A activity (D3L-based recruitment; Fig. 4c).

Although the C terminus of DNMT3L is able to dimerize with DNMT3A, we hypothesized that the ADD domain located in the N-terminal portion also plays an important role. Therefore full-length DNMT3L (D3L) was fused to either the N or the C terminus of dCas9 resulting in D3L-dCas9 and dCas9-D3L, respectively (Fig. 4d). Overexpression of DNMT3L in addition to KRAB-dCas9 and D3A-dCas9 yielded the strongest long-term repression (2.7-fold) at the *Snurf* promoter in Neuro2A cells (Fig. 4a). The combination of KRAB-dCas9 with dCas9-D3a3L resulted in long-term repression, but at a reduced level (1.4-fold) (Fig. 4a). Comparable repression (1.5-fold) was obtained when KRAB-dCas9 was combined with D3A-dCas9 and targetable DNMT3L fusions (D3L-dCas9 and dCas9-D3L; Fig. 4b). Ezh2-dCas9 also showed the strongest *Snurf* repression (threefold) when added with D3A-dCas9 and overexpressed D3L. On the other hand, Ezh2-dCas9 did not show any long-term repression when accompanied by dCas9-D3a3L or the combination of D3A-dCas9 and D3L-dCas9 (N-terminal D3L fusion). Thus, dCas9-D3L (C-terminal D3L fusion) was the only targetable version of DNMT3L capable of inducing long-term *Snurf* repression (1.8-fold) in combination with Ezh2-dCas9 and D3A-dCas9 (Fig. 4b). The same trend was observed at the *HER2* locus in HCT116 cells. Ezh2-dCas9 and D3A-dCas9 were only able to persistently repress *HER2* expression when combined with D3L overexpression or with the targetable C-terminal version dCas9-D3L (1.6 or 1.4-fold, respectively). Both the addition of dCas9-D3a3L and the combination of D3A-dCas9 with N-terminal D3L-dCas9 were not able to induce long-term repression, suggesting that full-length DNMT3L is necessary for long-term repression by Ezh2-dCas9 and D3A-dCas9, either due to recruitment of protein partners or to effects on activity due to conformational differences between the different constructs.

Overexpression of targetable DNA methyltransferase DNMT3A has recently raised concerns about off-target DNA methylation. The enzymatic activity of DNMT3A is enhanced by its dimerization partner DNMT3L, which does not display catalytic activity by itself. So far, we have introduced D3L by overexpression or by co-targeting with dCas9-D3L (Fig. 4a, b). We hypothesized that targeting of dCas9-D3L to an endogenous promoter is sufficient for recruitment of endogenous D3A to stably repress the target locus. To test this hypothesis, we co-transfected LNCaP cells with a combination of dCas9-D3L and either KRAB-dCas9 or Ezh2-dCas9 together with a gRNA pool targeted to the *SNURF* and *HER2* promoters. Long-term repression was determined 14 days after transfection and compared to dCas9 with no effector. Significant long-term repression was observed with dCas9-D3L and either KRAB-dCas9 or Ezh2-dCas9 with 1.8- or 1.3-fold repression of *SNURF* expression, respectively and 2.5- or twofold of *HER2* expression, respectively (Fig. 4c). We performed bisulfite sequencing to determine the methylation status achieved with the D3L-based recruitment approach at the *HER2* promoter (Fig. 4e). To directly compare this approach to the previously applied combinatorial treatment of KRAB-dCas9 or Ezh2-dCas9 with D3A-dCas9 and overexpressed D3L (Fig. 1d), we collected HER2− cells by FACS 4 days after co-transfection with three gRNAs targeting the *HER2* promoter in HCT116 cells. HER2− cells were expanded until sufficient amounts were available for bisulfite sequencing (10 days). Co-targeting of KRAB-dCas9, D3A-dCas9 and dCas9-D3L resulted in 22% methylation, while combinatorial treatment of KRAB-dCas9/D3A-dCas9 with overexpressed D3L increased methylation to 46%. When D3A-dCas9 was omitted, combinatorial treatment of KRAB-dCas9 or Ezh2-dCas9 with dCas9-D3L resulted in an average methylation of 20% and 29%, respectively (Fig. 4e). Surprisingly, comparable methylation levels (20%) were reached when dCas9-D3L itself was targeted to the *HER2* promoter. The *HER2* promoter region was unmethylated in the untreated control and in cells treated with dCas9 (no effector domain). Co-targeting of KRAB-dCas9, D3A-dCas9 and dCas9-D3L to the *HER2* promoter resulted in low levels of methylation (9%) suggesting that this approach is less efficient, which is also reflected in lower repressive ability (Fig. 4b).

Long-term repression facilitated by dCas9-D3L is advantageous for two reasons; (1) co-targeting requires only one epi-dCas9 (D3L fusion) instead of two components (D3A-dCas9 plus dCas9-D3L) for efficient long-term repression and (2) co-targeting with dCas9-D3L avoids overexpression of enzymatically active D3A and may therefore offer another strategy to limit off-target effects.

## Discussion

Although a variety of gene targeting tools exist that can robustly alter transcriptional activity for a short period, there is currently not a single tool or combination of tools that can universally invoke persistent epigenetic silencing of any desired gene in a predictable manner. There is a need for alternative hit-and-run epigenome editing tools that have a lasting effect in cells with only transient exposure to the tool itself. In this study, we observed that combinatorial treatment with Ezh2-dCas9 and DNA methylation (D3A-dCas9 and overexpressed D3L) was able to initiate long-term repression at two genomic loci (*SNURF*, *HER2*) in four different cell types. Although combinatorial KRAB-dCas9 treatment with D3A-dCas9 and overexpressed D3L was able to initiate long-term repression at most target genes tested, it failed to maintain persistence at *HER2* in HCT116 cells. We found that combinatorial KRAB-dCas9 treatment (KRAB + D3A + D3L), triggered a strong burst in H3K9me3 at the target locus, but the repressive H3K9me3 mark was completely lost at 24 days after treatment. The loss of H3K9me3 goes hand in hand with maintained hypermethylation of only one CpG probe in the *HER2* promoter, which was not sufficient for persistence and resulted in re-activation of *HER2* gene expression. On the other hand, combinatorial Ezh2-dCas9 treatment (Ezh2 + D3A + D3L) led to the establishment and maintenance of a repressive chromatin environment and long-term *HER2* repression. Both histone methylation (H3K27me3) and DNA methylation marks were maintained through approximately 57 cell divisions. DNA methylation did not stay restricted to the promoter region targeted by *HER2* gRNAs, but expanded to the adjacent CpG island covering a total of 1.25 kb.

Individual dCas9 fusions with epigenetic effector domains were in general not efficient in establishing epigenetic memory. There were a few exceptions that might have been cell type or locus specific. Similarly, Ezh2-dCas9 was only able to induce long-term repression in combination with D3A-dCas9 and D3L. In fact, we found that full-length D3L is required for Ezh2-dCas9 mediated long-term repression. The epi-dCas9 fusion with a hybrid methyltransferase containing a direct fusion of the C-terminal portion of DNMT3L to the catalytic domain of Dnmt3a (D3a3L; [53]) was unable to establish long-term epigenetic memory. Our data suggest that the N-terminus of DNMT3L containing the ADD domain, which interacts with the unmodified histone H3 tail [54], is important for establishing this interaction; the 3a3L fusion lacks this domain. Since the dCas9-D3a3L fusion is effective in DNA methylation, we hypothesize that DNA and histone methylation alone are not sufficient for long-term repression, but that the interaction between Ezh2 and DNMT3A/DNMT3L itself is important for establishment and maintenance of epigenetic memory. Ezh2 as well as EED have been shown to directly interact with DNMT3A. The N-terminal portion of Ezh2 has been shown to directly interact with the de novo DNA methyltransferases DNMT3A and DNMT3B and with the maintenance methyltransferase DNMT1.

Why might the establishment and inheritance of repressive chromatin states require both histone and DNA methyltransferases? In nature, a positive feedback loop between H3 lysine 9 (H3K9) methylation and DNA methylation has been well established and is a characteristic hallmark of mammalian heterochromatin [55–57]. Crosstalk between DNA methylation and H3K9 methylation is facilitated by the methyl-CpG-binding domain protein (MeCP2) as well as by heterochromatin protein 1 (HP1) [55, 57]. Heterochromatin marked by H3K9me3 and 5mC is bound by the KAP1/SETDB1(ESET) co-repressor complex, which is recruited by the Krueppel-associated box (KRAB) domain [18, 19]. Either DNA de-methylation or knockdown of KAP1 or SETDB1 lead to loss of DNA and H3K9 methylation and are accompanied by transcriptional derepression [58, 59]. The interplay between H3K27me3 and DNA methylation is less obvious, but nonetheless important for cell development and cellular identity. Unlike H3K9me3, H3K27me3 and DNA methylation are mutually exclusive marks at many genomic regions with high CpG density. However, a subset of promoter regions including bivalent gene promoters demonstrate crosstalk between H3K27me3 and DNA methylation [60]. Bivalent domains are found at promoters of developmentally regulated genes in stem cells and carry histone marks for both active and repressed chromatin (H3K4me3 and H3K27me3, respectively). Bivalently marked genes are silenced in undifferentiated stem cells, but considered poised for expression upon developmental cues [61]. Interestingly, H3K27me3 was reduced at nearly all bivalent promoters in mouse embryonic stem cells lacking DNA methyltransferases [60, 62–64]. Lower H3K27me3 occupancy was accompanied by reduced presence of PRC2 complex members Ezh2 and Suz12 [62, 63]. Effects on H3K27me3 were not only conferred by 5mC, but were also dependent on interactions with enzymatically active DNMT proteins (DNMT1, DNMT3A and DNMT3B; [65]. It has been proposed that DNA methylation at H3K27me3 marked regions leads to persistent gene repression and that this epigenetic memory may require interaction between the H3K27 methyltransferase EZH2 and DNA methyltransferase DNMT3A [65]. These studies indicate that DNA methylation plays an important role in the maintenance of normal promoter H3K27me3 patterns in a context-specific manner.

A general concern regarding off-target activity has been raised regarding using the active Cas9 nuclease for possible corrective treatment in human cells. Such off-target effects cause permanent changes to the DNA sequence. However, careful titration of Cas9 protein, protein engineering and careful design of gRNAs have given Cas9 the ability to function as a precise genome editing tool [66, 67]. Recently, concerns have been raised regarding dCas9-based epigenome editing (epi-dCas9) tools. Several reports have demonstrated high specificity of dCas9-KRAB [22, 68] and dCas9-DNMT3A [25]. However, some studies have demonstrated off-target activity of dCas9 fusions with DNMT3A [42, 43, 45]. Hypermethylation at single CpGs may, in fact, be genuine off-target events since they are reproducibly found in biological replicates. Notably, we do not observe robust off-target methylation under our experimental conditions. Differences in these studies can most likely be explained by the different experimental designs. Similar to Cas9 whose off-target cleavage activity increased with the amount of Cas9 enzyme present, off-target methylation is dependent on the dCas9-DNMT3A/3B concentration [43, 45]. Furthermore, we have demonstrated that dCas9-D3L is sufficient to initiate long-term silencing by Ezh2-dCas9 or KRAB-dCas9. We also found that targeting D3L alone can methylate the *HER2* locus, but the implications for long-term silencing and spreading of DNA methylation have not yet been explored. These findings suggest that targeting dCas9-D3L, which lacks enzymatic activity itself, recruits endogenous DNA methyltransferase DNMT3A. This may offer an advantage compared to overexpression of an active enzyme (D3A) in limiting off-target activity. On the other hand, overexpression of D3L may act as a sponge for endogenous D3A, potentially causing misregulation by hypomethylation of off-target genes. Future studies should carefully examine these possibilities.

We then explored two strategies that would allow recruitment of multiple epi-dCas9 fusions, while limiting the number of gRNAs in order to reduce potential off-target sites. Surprisingly, we found that a single gRNA induced persistent silencing as well as a pool of 3 gRNA when targeting two different epi-dCas9 effectors (KRAB-dCas9/D3A-dCas9 plus D3L) to the *Snurf* promoter. Similar observations have been reported [25] but only for a subset of gRNAs. They reported that a pool of gRNA was as good or better than individual gRNAs when co-targeting multiple epi-dCas9 fusions. Therefore, we don’t expect that this observation is generalizable to any gRNA, but it does mean that different epi-dCas9 molecules can interact dynamically through a single gRNA or that different epi-dCas9 s aggregate through gRNA independent interactions. We also tested RNA-mediated recruitment of epigenetic repressor domains to increase the combinatorial capacity of the epi-dCas9 system while maintaining a reduced number of gRNAs. However, recruitment of MCP-Ezh2 by MS2 stem loop-containing gRNAs did not result in repression of the target locus. It is possible that the relatively large size of Ezh2 (85 kDa) destabilizes the dCas9 complex with the target site. MCP-KRAB was able to repress the target locus, but with less efficiency than the direct dCas9-KRAB fusion. It is possible that the altered structure of the MS2-gRNAs, as compared to standard gRNAs, results in reduced recruitment of dCas9 or less stable dCas9/target interactions, leading to lower repressive capacity. However, we do note that the MS2-based recruitment approach has worked quite efficiently for gene activation [33, 49–52]. Therefore, it is possible that different experimental parameters could lead to stronger repressive capacity using this system.

There has been a significant effort to determine features that make a specific genomic site amenable to cleavage by the RNA-guided nuclease Cas9. There is some evidence for sequence-specific context [69], but, more importantly, cleavage by Cas9 is significantly inhibited by nucleosomes in vivo [70–72]. The field of epigenome editing faces similar challenges that can only partially be explained by sequence features or the presence of nucleosomes. Since most epi-dCas9 fusions are targeted to DNase hypersensitivity sites, the variation of success must arise from other features. The effect of targeted epigenetic reprograming might be influenced by factors such as epigenetic marks, three-dimensional interactions (e.g., between a promoter and an enhancer, or localization of the DNA region to a subnuclear compartment such as a transcriptional factory), and initial expression levels, which may be locus and cell-type dependent. A systematic study encompassing a large set of loci is needed to identify determinants that make a locus amenable to engineering persistent silencing to ensure more predictable outcomes. In addition, engineering epigenetic memory may depend on cellular factors that vary in different cell types. Some differences may be related to the use of dividing versus non-dividing cells [25, 42], more studies are needed to establish guidelines that can predict which combination of targetable epigenetic effectors can establish and maintain an epigenetic switch at any desired locus and cell type.

It should be possible to treat epigenetic disorders by altering the epigenetic information at specific loci. To avoid the need for lifelong expression of an exogenous modifier protein, it is critical to establish a persistent epigenetic state, as seen in nature. However, there is currently no single or combination of epigenetic targeting tools that can induce epigenetic memory at any desired locus in any cell type which targeting cocktail is most efficient is cell type dependent, revealing a not yet understood cell-specific context for establishment of epigenetic memory [25, 26, 42]. Although an epi-dCas9 cocktail containing KRAB, DNMT3A, and DNMT3L was able to repress endogenous genes to various degrees (*BM2, IFNAR1* and *VEGFA*) [25], the same cocktail was unable to maintain repression at the *HER2* locus in HCT116 cells [26]. Targeted DNA methylation facilitated through use of only DNMT3A has shown modest long-term repression in some but not all reporter cell lines tested [25, 73]. Similarly, a dCas9 fusion with DNMT3A-DNMT3L was able to induce long-term repression at some, but not all, targeted genes in primary myoepithelial cells [42]. Although many writers and readers of histone modifications have been identified (reviewed in [8]), it remains an open question how they collaborate to establish genome-wide chromatin patterns and epigenetic memory in different chromatin and cellular contexts.

## Conclusions

Epigenetic changes in nature are associated with long-term activation or silencing of gene transcription. With the ability to target such changes in epigenetic information, it should be possible to achieve the same long-term effects as are observed in nature. However, the observed effects of epigenetic editing are often of limited magnitude and duration. One hypothesis is that there is a lack of sufficient understanding of the initial epigenetic state of the target gene, and how that specific state is affected by the editing tools. The work reported here demonstrates (a) a clear difference between the histone methyltransferase requirements for short- and long-term silencing, and (b) that those requirements can be different for different target genes. These results represent a significant expansion beyond the prevailing paradigm that persistent silencing can be best achieved by a combination of KRAB, DNMT3A and DNMT3L [25]. Further studies are needed to obtain a more detailed understanding of the mechanisms underlying these observed differences and to enable predictable targeted control of epigenetic effects at individual loci that are similar to those observed in nature.

## Additional files

**Additional file 1: Figure S1.** Amino acid sequences of dCas9- and MCP fusion proteins.

**Additional file 2: Table S1.** gRNA target sequences, predicted off-target sites and oligonucleotide sequences of RT-qPCR primers.

**Additional file 3: Figure S2.** Evaluation of HER2 protein levels and all-or-none epigenetic silencing.

**Additional file 4: Figure S3.** Long-term repression of *TRPM4* locus in C42B cells.

**Additional file 5: Figure S4.** Reproducibility of H3K27ac ChIP-seq replicates.

**Additional file 6: Table S2.** Lists of H3K27ac ChIP-seq peaks.

**Additional file 7: Figure S5.** H3K27ac ChIP-seq analysis after hit-and-run epigenetic editing.

**Additional file 8: Figure S6.** Reproducibility of global DNA methylation analysis after hit-and-run epigenetic editing.

**Additional file 9: Table S3.** Lists of hypermethylated CpG probes and hypermethylated gene promoters (>3 probes).

**Additional file 10: Figure S7.** Alternative epi-dCas9 recruitment strategies while maintaining a reduced number of gRNAs.

## Abbreviations

DNMT: DNA methyltransferase
D3A: DNA methyltransferase 3A
D3L: DNA methyltransferase 3L
ChIP: chromatin immunoprecipitation
