# Supplementary material for: Ezh2-dCas9 and KRAB-dCas9 enable engineering of epigenetic memory in a context-dependent manner
Source: Epigenetics Chromatin. 2019 May 3;12:26. doi: 10.1186/s13072-019-0275-8 (PMC6498470; doi:10.1186/s13072-019-0275-8)
Supplement: Supplementary file 1 — Additional file 1: Figure S1. Amino acid sequences of dCas9- and MCP fusion proteins. [file 13072_2019_275_MOESM1_ESM.pdf]

Supplemental Figure S1: Amino acid sequences of dCas9- and MCP-fusion proteins.

D3L-dCas9

NLS (SV40): aa 2-9
3X Flag: aa 15-36
D3L human DNMT3L (NP_787063): aa 47-432
dCas9 (D10A,H840A): aa 453-1820
NLS (Neoplasmin): aa 1846-1861

MPKKKRKVGGSGGSDYKDHDGDYKDHDIDYKDDDDKGGGSGGGSGTMAAIPALDPEAEPSMDV
ILVGSSELSSSVSPGTGRDLIAYEVKANQRNIEDICICCGSLQVHTQHPLFEGGICAPCKDKF
LDALFLYDDDGYQSYCSICCSGETLLICGNPDCTRCYCFECVDSLVGPGTSGKVHAMSNWVCY
LCLPSSRSGLLQRRRKWRSQLKAFYDRESENPLEMFETVPVWRRQPVRVLSLFEDIKKELTSL
GFLESGSDPGQLKHVVDVTDTVRKDVEEWGPFDLVYGATPPLGHTCDRPPSWYLFQFHRLLQY
ARPKPGSPRPFFWMFVDNLVLNKEDLDVASRFLEMEPVTIPDVHGGSLQNAVRVWSNIPAIRS
RHWALVSEEELSLLAQNKQSSKLAAKWPTKLVKNCFLPLREYFKYFSTELTSSLSTGGSGGSG
GSGGSGGSGRPMDKKYSIGLAIGTNSVGWAVITDEYKVPSKKFKVLGNTDRHSIKKNLIGALL
FDSGETAEATRLKRTARRRYTRRKNRICYLQEIFSNEMAKVDDSFFHRLEESFLVEEDKKHER
HPIFGNIVDEVAYHEKYPTIYHLRKKLVDSTDKADLRLIYLALAHMIKFRGHFLIEGDLNPDN
SDVDKLFIQLVQTYNQLFEENPINASGVDAKAILSARLSKSRRLENLIAQLPGEKKNGLFGNL
IALSLGLTPNFKSNFDLAEDAKLQLSKDTYDDDLDNLLAQIGDQYADLFLAAKNLSDAILLSD
ILRVNTEITKAPLSASMIKRYDEHHQDLTLLKALVRQQLPEKYKEIFFDQSKNGYAGYIDGGA
SQEEFYKFIKPILEKMDGTEELLVKLNREDLLRKQRTFDNGSIPHQIHLGELHAILRRQEDFY
PFLKDNREKIEKILTFRIPYYVGPLARGNSRFAWMTRKSEETITPWNFEEVVDKGASAQSFIE
RMTNFDKNLPNEKVLPKHSLLYEYFTVYNELTKVKYVTEGMRKPAFLSGEQKKAIVDLLFKTN
RKVTVKQLKEDYFKKIECFDSVEISGVEDRFNASLGTYHDLLKIIKDKDFLDNEENEDILEDI
VLTLTLFEDREMIEERLKTYAHLFDDKVMKQLKRRRYTGWGRLSRKLINGIRDKQSGKTILDF
LKSDGFANRNFMQLIHDDSLTFKEDIQKAQVSGQGDSLHEHIANLAGSPAIKKGILQTVKVVD
ELVKVMGRHKPENIVIEMARENQTTQKGQKNSRERMKRIEEGIKELGSQILKEHPVENTQLQN
EKLYLYYLQNGRDMYVDQELDINRLSDYDVDAIVPQSFLKDDSIDNKVLTRSDKNRGKSDNVP
SEEVVKKMKNYWRQLLNAKLITQRKFDNLTKAERGGLSELDKAGFIKRQLVETRQITKHVAQI
LDSRMNTKYDENDKLIREVKVITLKSKLVSDFRKDFQFYKVREINNYHHAHDAYLNAVVGTAL
IKKYPKLESEFVYGDYKVYDVRKMIAKSEQEIGKATAKYFFYSNIMNFFKTEITLANGEIRKR
PLIETNGETGEIVWDKGRDFATVRKVLSMPQVNIVKKTEVQTGGFSKESILPKRNSDKLIARK
KDWDPKKYGGFDSPTVAYSVLVVAKVEKGKSKKLKSVKELLGITIMERSSFEKNPIDFLEAKG
YKEVKKDLIIKLPKYSLFELENGRKRMLASAGELQKGNELALPSKYVNFLYLASHYEKLKGSP
EDNEQKQLFVEQHKHYLDEIIEQISEFSKRVILADANLDKVLSAYNKHRDKPIREQAENIIHL
FTLTNLGAPAAFKYFDTTIDRKRYTSTKEVLDATLIHQSITGLYETRIDLSQLGGDGGSGGSG
GSGGSGGSASGGGSGGGSKRPAATKKAGQAKKKKGGSGSGATNFSLLKQA

dCas9-D3L

NLS (SV40): aa 2-9
3X Flag: aa 15-36
dCas9 (D10A,H840A): aa 65-1432
D3L human DNMT3L (NP_787063): aa 1450-1835
NLS (Neoplasmin): aa 1845-1860

MPKKKRKVGGSGGSDYKDHDGDYKDHDIDYKDDDDKGGGSGGGSGTGGSGGSGGSGGSGGSGR
PMDKKYSIGLAIGTNSVGWAVITDEYKVPSKKFKVLGNTDRHSIKKNLIGALLFDSGETAEAT
RLKRTARRRYTRRKNRICYLQEIFSNEMAKVDDSFFHRLEESFLVEEDKKHERHPIFGNIVDE
VAYHEKYPTIYHLRKKLVDSTDKADLRLIYLALAHMIKFRGHFLIEGDLNPDNSDVDKLFIQL
VQTYNQLFEENPINASGVDAKAILSARLSKSRRLENLIAQLPGEKKNGLFGNLIALSLGLTPN
FKSNFDLAEDAKLQLSKDTYDDDLDNLLAQIGDQYADLFLAAKNLSDAILLSDILRVNTEITK
APLSASMIKRYDEHHQDLTLLKALVRQQLPEKYKEIFFDQSKNGYAGYIDGGASQEEFYKFIK
PILEKMDGTEELLVKLNREDLLRKQRTFDNGSIPHQIHLGELHAILRRQEDFYPFLKDNREKI
EKILTFRIPYYVGPLARGNSRFAWMTRKSEETITPWNFEEVVDKGASAQSFIERMTNFDKNLP
NEKVLPKHSLLYEYFTVYNELTKVKYVTEGMRKPAFLSGEQKKAIVDLLFKTNRKVTVKQLKE
DYFKKIECFDSVEISGVEDRFNASLGTYHDLLKIIKDKDFLDNEENEDILEDIVLTLTLFEDR
EMIEERLKTYAHLFDDKVMKQLKRRRYTGWGRLSRKLINGIRDKQSGKTILDFLKSDGFANRN
FMQLIHDDSLTFKEDIQKAQVSGQGDSLHEHIANLAGSPAIKKGILQTVKVVDELVKVMGRHK
PENIVIEMARENQTTQKGQKNSRERMKRIEEGIKELGSQILKEHPVENTQLQNEKLYLYYLQN
GRDMYVDQELDINRLSDYDVDAIVPQSFLKDDSIDNKVLTRSDKNRGKSDNVPSEEVVKKMKN
YWRQLLNAKLITQRKFDNLTKAERGGLSELDKAGFIKRQLVETRQITKHVAQILDSRMNTKYD
ENDKLIREVKVITLKSKLVSDFRKDFQFYKVREINNYHHAHDAYLNAVVGTALIKKYPKLESE
FVYGDYKVYDVRKMIAKSEQEIGKATAKYFFYSNIMNFFKTEITLANGEIRKRPLIETNGETG
EIVWDKGRDFATVRKVLSMPQVNIVKKTEVQTGGFSKESILPKRNSDKLIARKKDWDPKKYGG
FDSPTVAYSVLVVAKVEKGKSKKLKSVKELLGITIMERSSFEKNPIDFLEAKGYKEVKKDLII
KLPKYSLFELENGRKRMLASAGELQKGNELALPSKYVNFLYLASHYEKLKGSPEDNEQKQLFV
EQHKHYLDEIIEQISEFSKRVILADANLDKVLSAYNKHRDKPIREQAENIIHLFTLTNLGAPA
AFKYFDTTIDRKRYTSTKEVLDATLIHQSITGLYETRIDLSQLGGDGGSGGSGGSGGSGGSAS
MAAIPALDPEAEPSMDVILVGSSELSSSVSPGTGRDLIAYEVKANQRNIEDICICCGSLQVHT
QHPLFEGGICAPCKDKFLDALFLYDDDGYQSYCSICCSGETLLICGNPDCTRCYCFECVDSLV
GPGTSGKVHAMSNWVCYLCLPSSRSGLLQRRRKWRSQLKAFYDRESENPLEMFETVPVWRRQP
VRVLSLFEDIKKELTSLGFLESGSDPGQLKHVVDVTDTVRKDVEEWGPFDLVYGATPPLGHTC
DRPPSWYLFQFHRLLQYARPKPGSPGPFFWMFVDNLVLNKEDLDVASRFLEMEPVTIPDVHGG
SLQNAVRVWSNIPAIRSRHWALVSEEELSLLAQNKQSSKLAAKWPTKLVKNCFLPLREYFKYF
STELTSSLSGGGSGGGSKRPAATKKAGQAKKKKGGSGSGATNFSLLKQAGDVEENPGPAAA

2xMCP-KRAB

2xMCP: aa 1-236
KRAB (NP_056209.2, aa12-85): aa 241-312

MASNFTQFVLVDNGGTGDVTVAPSNFANGIAEWISSNSRSQAYKVTCSVRQSSAQNRKYTIKV
EVPKGAWRSYLNMELTIPIFATNSDCELIVKAMQGLLKDGNPIPSAIAANSGIYAMASNFTQF
VLVDNGGTGDVTVAPSNFANGIAEWISSNSRSQAYKVTCSVRQSSAQNRKYTIKVEVPKGAWR
SYLNMELTIPIFATNSDCELIVKAMQGLLKDGNPIPSAIAANSGIYADSRMVTFKDVFVDFTR
EEWKLLDTAQQIVYRNVMLENYKNLVSLGYQLTKPDVILRLEKGEEPWLVEREIHQETHP

2xMCP-Ezh2

2xMCP
Ezh2 (NP_031997.2, aa 1-746)

MASNFTQFVLVDNGGTGDVTVAPSNFANGIAEWISSNSRSQAYKVTCSVRQSSAQNRKYTIKV
EVPKGAWRSYLNMELTIPIFATNSDCELIVKAMQGLLKDGNPIPSAIAANSGIYAMASNFTQF
VLVDNGGTGDVTVAPSNFANGIAEWISSNSRSQAYKVTCSVRQSSAQNRKYTIKVEVPKGAWR
SYLNMELTIPIFATNSDCELIVKAMQGLLKDGNPIPSAIAANSGIYADSRGGGSGGGSGTMGQ
TGKKSEKGPVCWRKRVKSEYMRLRQLKRFRRADEVKTMFSSNRQKILERTETLNQEWKQRRIQ
PVHIMTSVSSLRGTRECSVTSDLDFPAQVIPLKTLNAVASVPIMYSWSPLQQNFMVEDETVLH
NIPYMGDEVLDQDGTFIEELIKNYDGKVHGDRECGFINDEIFVELVNALGQYNDDDDDDDGDD
PDEREEKQKDLEDNRDDKETCPPRKFPADKIFEAISSMFPDKGTAEELKEKYKELTEQQLPGA
LPPECTPNIDGPNAKSVQREQSLHSFHTLFCRRCFKYDCFLHPFHATPNTYKRKNTETALDNK
PCGPQCYQHLEGAKEFAAALTAERIKTPPKRPGGRRRGRLPNNSSRPSTPTISVLESKDTDSD
REAGTETGGENNDKEEEEKKDETSSSSEANSRCQTPIKMKPNIEPPENVEWSGAEASMFRVLI
GTYYDNFCAIARLIGTKTCRQVYEFRVKESSIIAPVPTEDVDTPPRKKKRKHRLWAAHCRKIQ
LKKDGSSNHVYNYQPCDHPRQPCDSSCPCVIAQNFCEKFCQCSSECQNRFPGCRCKAQCNTKQ
CPCYLAVRECDPDLCLTCGAADHWDSKNVSCKNCSIQRGSKKHLLLAPSDVAGWGIFIKDPVQ
KNEFISEYCGEIISQDEADRRGKVYDKYMCSFLFNLNNDFVVDATRKGNKIRFANHSVNPNCY
AKVMMVNGDHRIGIFAKRAIQTGEELFFDYRYSQADALKYVGIEREMEIP
