# Supplementary material for: Ezh2-dCas9 and KRAB-dCas9 enable engineering of epigenetic memory in a context-dependent manner
Source: Epigenetics Chromatin. 2019 May 3;12:26. doi: 10.1186/s13072-019-0275-8 (PMC6498470; doi:10.1186/s13072-019-0275-8)
Supplement: Supplementary file 3 — Additional file 3: Figure S2. Evaluation of HER2 protein levels and all-or-none epigenetic silencing. [file 13072_2019_275_MOESM3_ESM.pdf]

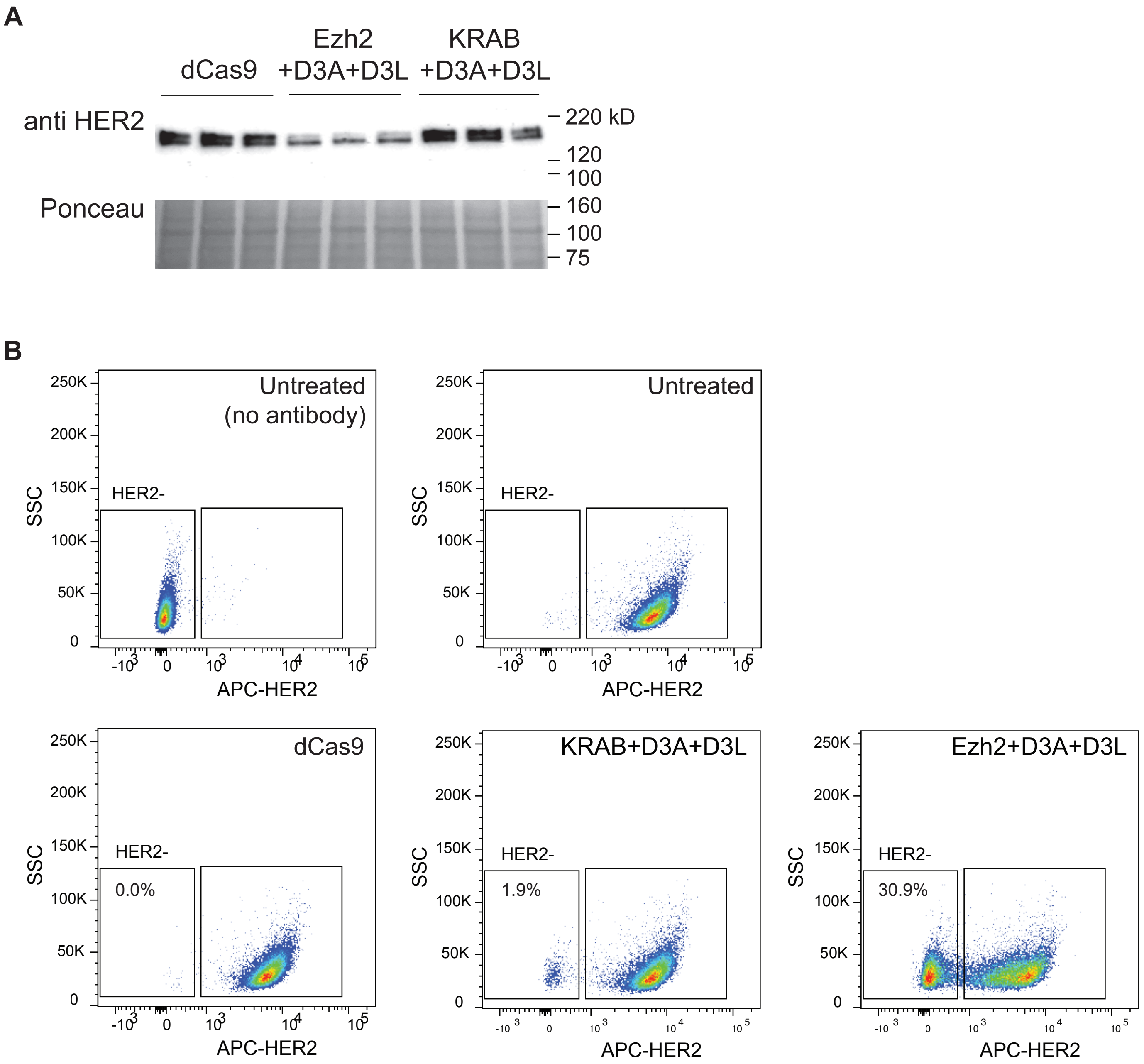


Supplemental Figure S2: Evaluation of HER2 protein levels and all-or-none epigenetic silencing. A. Western blot analysis of HER2 protein levels. HER2 protein levels were determined in HCT116 cells 40 days after transfection with dCas9 no ED, Ezh2-dCas9 or KRAB-dCas9 cocktails with D3A-dCas9 and D3L. Ponceau S staining is provided as a protein loading control. B. Representative flow cytometry data to identify HER2- and HER2+ subpopulations in HCT116 cells 50 days after collection of HER2 negative cells ( see Fig.1B) treated with Ezh2+D3A+D3L (Ezh2-dCas9, D3A-dCas9 and overexpressed D3L) or KRAB+D3A+D3L (KRAB-dCas9, D3A-dCas9 and overexpressed D3L) in the presence of three gRNAs targeting the HER2 promoter. HER2- cells are indicated. Untreated HCT116 cells were used to identify HER2+ population and HCT116 cells without antibody incubation served as a negative control. Cells treated with dCas9 without effector domain were used to evaluate the effect of combinatorial treatment with indicated epi-dCas9 fusions.
