# Supplementary material for: Ezh2-dCas9 and KRAB-dCas9 enable engineering of epigenetic memory in a context-dependent manner
Source: Epigenetics Chromatin. 2019 May 3;12:26. doi: 10.1186/s13072-019-0275-8 (PMC6498470; doi:10.1186/s13072-019-0275-8)
Supplement: Supplementary file 4 — Additional file 4: Figure S3. Long-term repression of TRPM4 locus in C42B cells. [file 13072_2019_275_MOESM4_ESM.pdf]

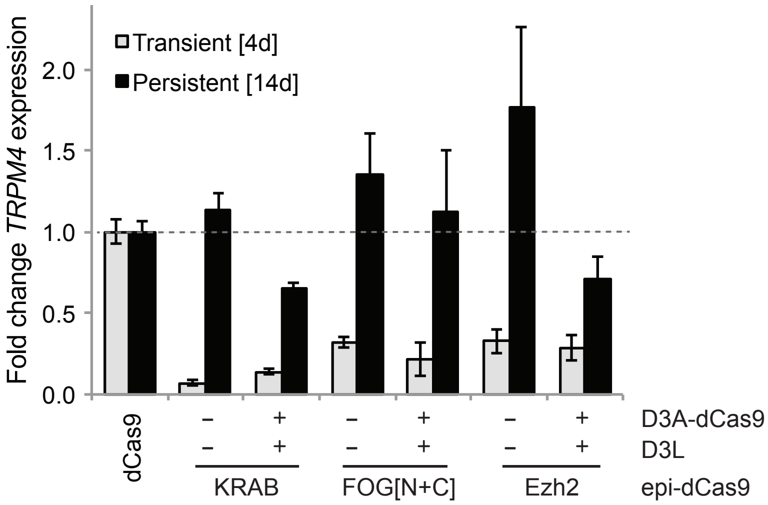

Supplemental Figure S3: Long-term repression of the *TRPM4* locus in C42B cells. RT-qPCR of endogenous *TRPM4* mRNA levels 4d (transient) and 14d (long-term) after co-transfection of plasmids expressing four gRNAs targeted to the *TRPM4* promoter and the indicated epi-dCas9 fusions individually or in combination with D3A-dCas9 and D3L. *TRPM4* mRNA levels were compared to dCas9 with no ED (n = 2; mean ± SEM).
