# Supplementary material for: Ezh2-dCas9 and KRAB-dCas9 enable engineering of epigenetic memory in a context-dependent manner
Source: Epigenetics Chromatin. 2019 May 3;12:26. doi: 10.1186/s13072-019-0275-8 (PMC6498470; doi:10.1186/s13072-019-0275-8)
Supplement: Supplementary file 5 — Additional file 5: Figure S4. Reproducibility of H3K27ac ChIP-seq replicates. [file 13072_2019_275_MOESM5_ESM.pdf]

A

	# Peaks
dCas9 rep1	57,339
dCas9 rep2	54,651
Ezh2+D3A+D3L rep1	56,494
Ezh2+D3A+D3L rep2	48,127

B

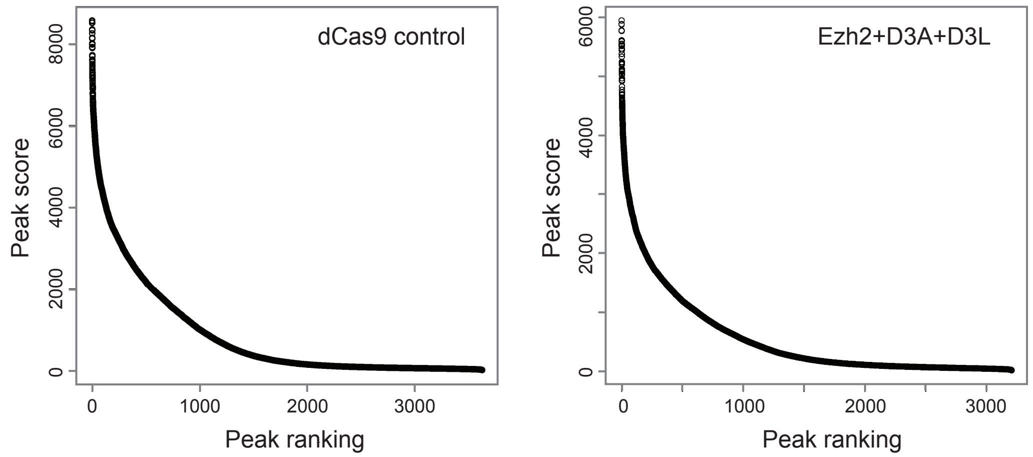

C

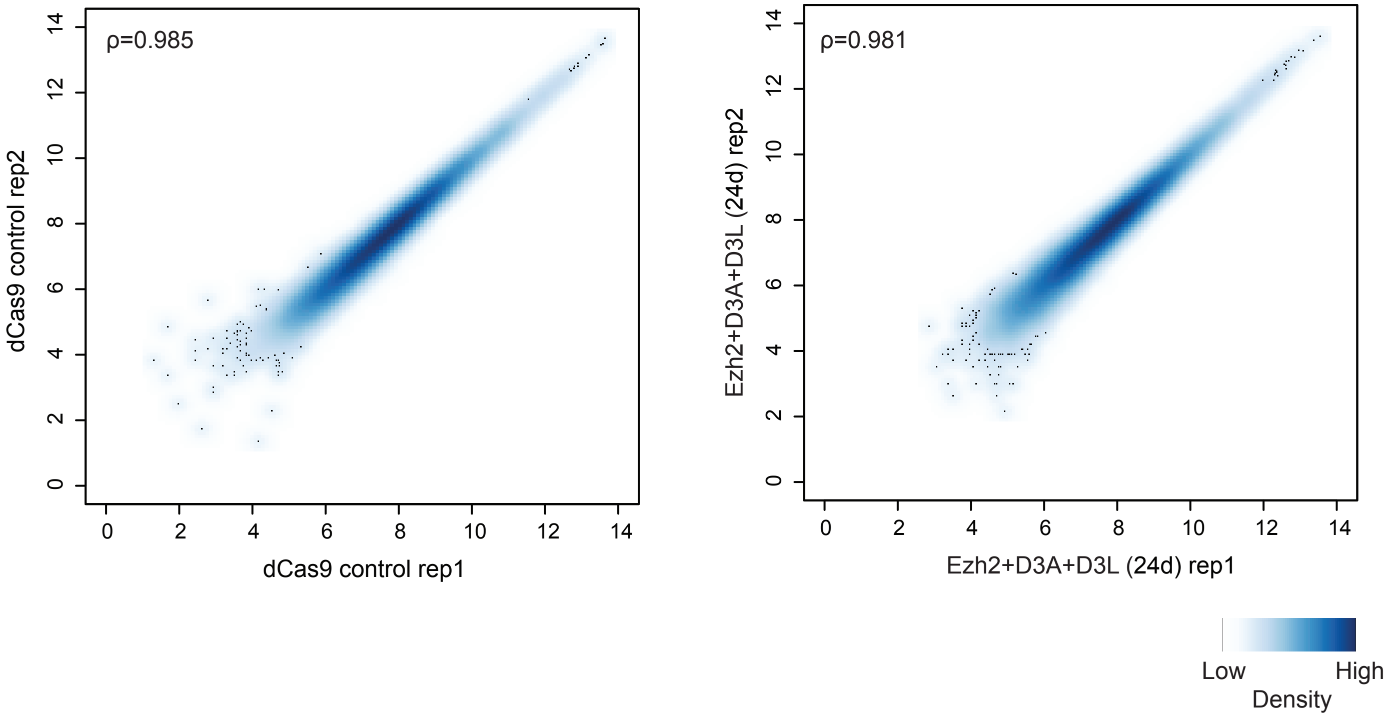

Supplemental Figure S4: Reproducibility of H3K27ac ChIP-seq replicates. ChIP-seq experiments were performed in HCT116 cells 24 days after transfection with plasmids expressing Ezh2-dCas9, D3A-dCas9 and D3L in the presence of three gRNAs targeting the *HER2* promoter. ChIP-seq of dCas9 with no ED was used as a control. **A.** Number of H3K27ac peaks called in two independent biological replicates using MACS2. **B.** Peak score from MACS for reproducible H3K27ac peaks. **C.** Scatterplot of peak scores for independent biological replicates.
