# Supplementary material for: Ezh2-dCas9 and KRAB-dCas9 enable engineering of epigenetic memory in a context-dependent manner
Source: Epigenetics Chromatin. 2019 May 3;12:26. doi: 10.1186/s13072-019-0275-8 (PMC6498470; doi:10.1186/s13072-019-0275-8)
Supplement: Supplementary file 7 — Additional file 7: Figure S5. H3K27ac ChIP-seq analysis after hit-and-run epigenetic editing. [file 13072_2019_275_MOESM7_ESM.pdf]

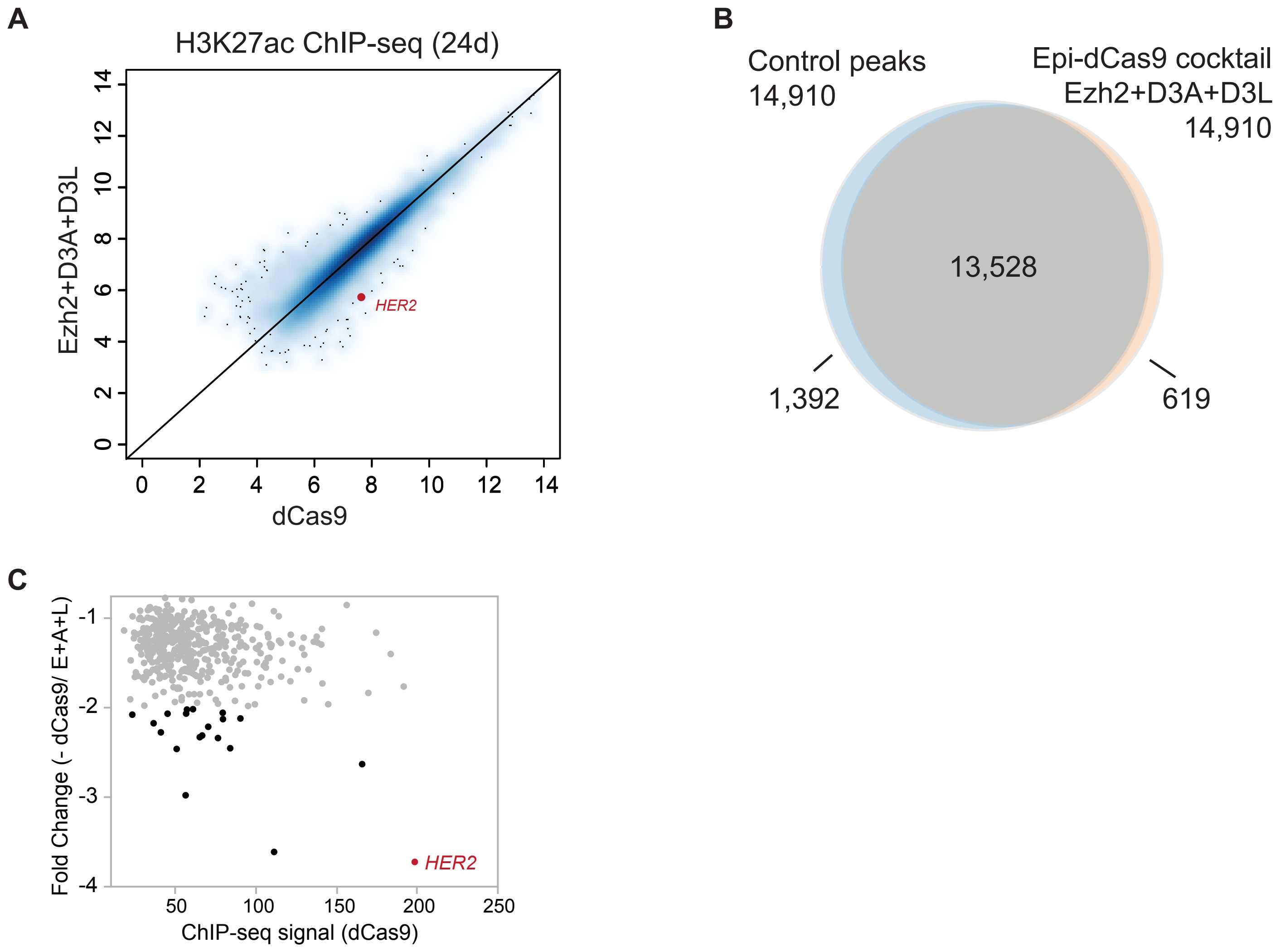

Supplemental Figure S5: H3K27ac ChIP-seq analysis after hit-and-run epigenetic editing. **A.** Density scatter plot of H3K27ac ChIP-seq signal in HCT116 cells 24 days after treatment with Ezh2-dCas9, D3A-dCas9 and D3L in comparison to dCas9 control treated cells. Target site in the HER2 promoter is indicated with a red dot. **B.** Overlap analysis of H3K27ac peaks from cells treated with Ezh2 plus D3A and D3L with control cells treated with dCas9 without ED. **C.** The fold-change (-dCas9/Ezh2+D3A+D3L) of peak scores is plotted against the ChIP-seq signal in the dCas9 control sample. ChIP-seq peaks with >2 fold change are represented as black dots. *HER2* target site is indicated with a red dot.
