# Supplementary material for: Ezh2-dCas9 and KRAB-dCas9 enable engineering of epigenetic memory in a context-dependent manner
Source: Epigenetics Chromatin. 2019 May 3;12:26. doi: 10.1186/s13072-019-0275-8 (PMC6498470; doi:10.1186/s13072-019-0275-8)
Supplement: Supplementary file 8 — Additional file 8: Figure S6. Reproducibility of global DNA methylation analysis after hit-and-run epigenetic editing. [file 13072_2019_275_MOESM8_ESM.pdf]

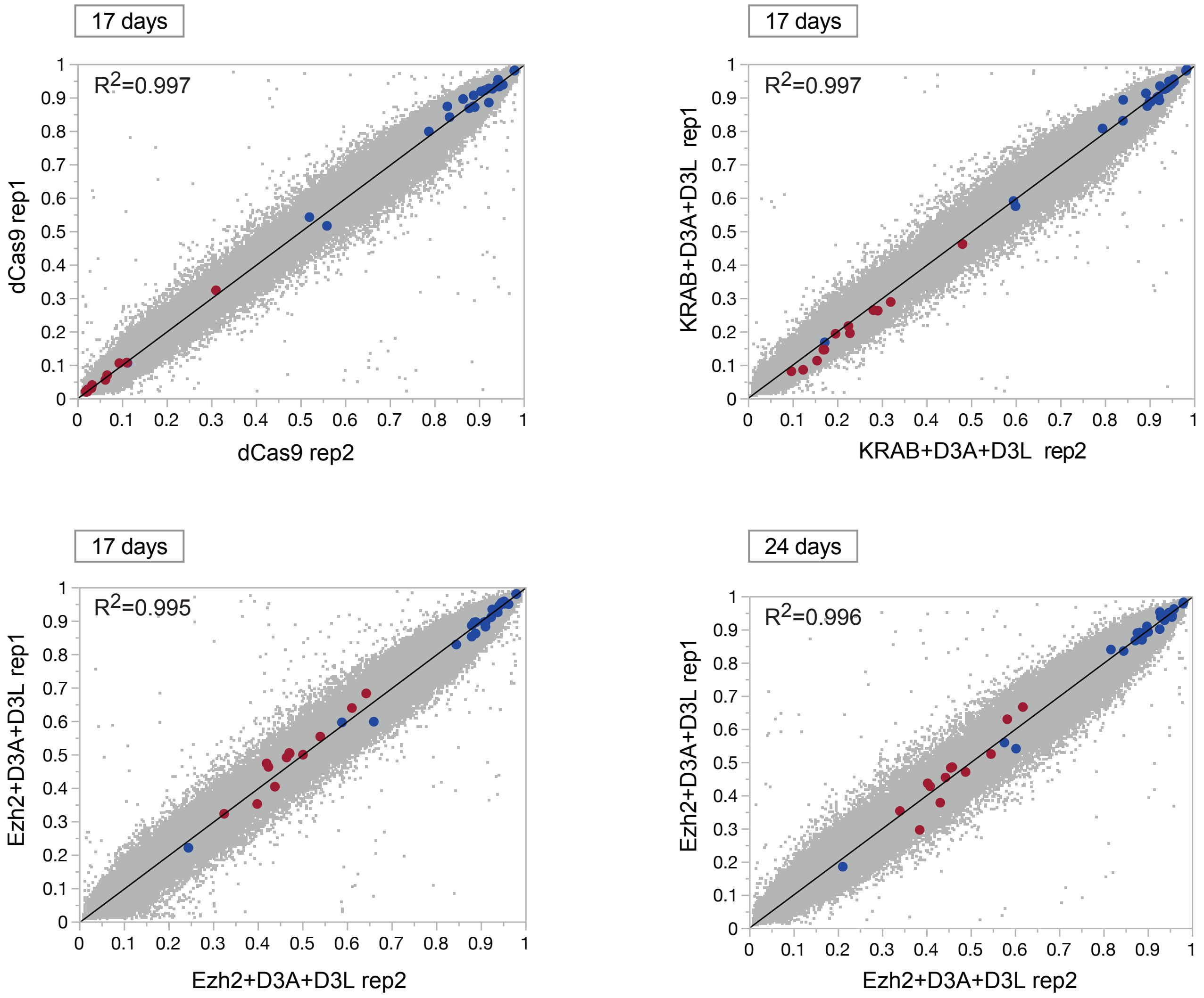

Supplemental Figure S6: Reproducibility of global DNA methylation analysis after hit-and-run epigenetic editing. DNA methylation level of ~865,000 individual CpG probes was determined in two independent biological replicates using the Infinium Human MethylationEPIC BeadChip (Illumina). Scatterplots demonstrate high reproducibility between biological replicates. HCT116 cells treated with KRAB+D3A +D3L and dCas9 control cells were evaluated 17 days after transfection. Methylation levels in cells treated with Ezh2+D3A+D3L were measured 17 and 24 days after transfection. *HER2* CpG probes in promoter region (5'UTR, TSS200, and TSS1500) are shown in red, all other CpG probes related to *HER2* (exon, body, 3'UTR) are shown in blue.
