# Supplementary material for: Ezh2-dCas9 and KRAB-dCas9 enable engineering of epigenetic memory in a context-dependent manner
Source: Epigenetics Chromatin. 2019 May 3;12:26. doi: 10.1186/s13072-019-0275-8 (PMC6498470; doi:10.1186/s13072-019-0275-8)
Supplement: Supplementary file 10 — Additional file 10: Figure S7. Alternative epi-dCas9 recruitment strategies while maintaining a reduced number of gRNAs. [file 13072_2019_275_MOESM10_ESM.pdf]

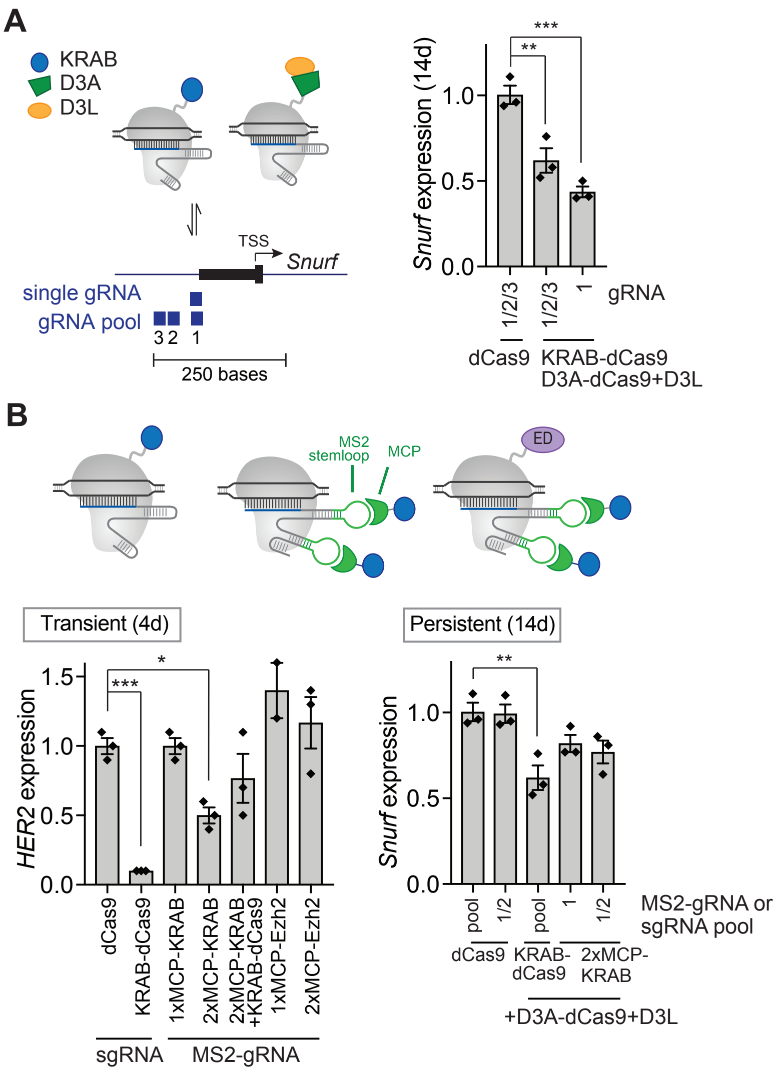

Supplemental Figure S7: Alternative epi-dCas9 recruitment strategies while maintaining a reduced number of gRNAs. A. Diagram depicting one or three gRNAs for targeting multiple epi-dCas9 fusions. *Snurf* mRNA levels were determined by RT-qPCR after co-transfection of plasmids expressing KRAB-dCas9, D3A-dCas9 and D3L with one or three gRNAs in Neuro2A cells and compared to dCas9 with no ED were determined (Dunnett’s test, **P<0.01, **P<0.001; n=3; mean ± SEM). B. Schematic of RNA based recruitment. dCas9 is recruited to gRNAs containing two MS2 stemloops allowing recruitment of an effector domain (KRAB) fused to MCP and combinatorial treatment by using a dCas9 fused to another effector domain (ED). Transient repression at the *HER2* locus in HCT116 cells was determined by RT-qPCR 4 days after co-transfection of indicated epi-dCas9 or MCP-ED fusions with sgRNAs or MS2-gRNAs, respectively (Dunnett’s test, *P<0.05, **P<0.01, **P<0.001; n=3; mean ± SEM). Long-term repression of Snurf in Neuro2A cells was evaluated by RT-qPCR 14 days after co-transfection of KRAB-dCas9 with gRNAs or a mix of dCas9, 2xMCP-KRAB with MS2-gRNAs. *Snurf* mRNA levels were compared to dCas9 with no ED (Dunnett’s test, *P<0.05, **P<0.01, **P<0.001; n=3; mean ± SEM).
